# Gap-free comparative genomics uncover virulence factors for Fusarium wilt of watermelons

**DOI:** 10.1371/journal.ppat.1013455

**Published:** 2025-08-25

**Authors:** Dilay Hazal Ayhan, Huan Wang, Lili Zhang, Guan Wang, Shu Yi, Dian Meng, Lifang Xue, Xin Geng, Zhijun Kong, Xinrui Wang, Lu Wang, Qingxian Yang, Xiangfeng Wang, Yun Deng, Xingping Zhang, Li Guo

**Affiliations:** 1 Shandong Key Laboratory of Precision Molecular Crop Design and Breeding, Peking University Institute of Advanced Agricultural Sciences, Shandong Laboratory of Advanced Agricultural Sciences in Weifang, Weifang, China; 2 Graduate School of Natural and Applied Sciences, Acıbadem University, Istanbul, Türkiye; 3 School of Agriculture, Ningxia University, Yinchuan, China; Chinese Academy of Sciences, CHINA

## Abstract

Watermelon (*Citrullus lanatus* L.) is a globally important fruit crop, yet it is susceptible to devastating diseases such as vascular wilt caused by *Fusarium oxysporum* f. sp. *niveum* (Fon), with limited control options. Fon rapidly evolves to overcome host resistance, constantly threatening production through new pathogenic races. High-quality genomic resources are key to understanding the molecular mechanisms underlying Fon virulence evolution for disease management. Here, we *de novo* assembled and annotated gapless genomes of three isolates affiliated with different physiological races of Fon (race 1, 2, and 3), and dissected the mechanisms behind their distinctive virulence through comparative genomics and transcriptomics. Core and accessory chromosomes in Fon were identified, where each race-affiliated isolate carried a unique set of accessory chromosomes or regions. Comparative transcriptomics of Fon infection revealed distinctive temporal patterns of gene expression even among core gene families, particularly those related to cell wall degradation enzymes. Effectoromic prediction and comparative analysis in three gap-free genomes identified 13 FonR3-specific effectors (FonR3SEs), one (FonR3SE1) of which was a critical virulence factor of FonR3 on watermelon as demonstrated via functional experiments. These gap-free genome assemblies and FonR3SEs provide valuable resources for studying Fon pathobiology and evolution and improving development of disease control strategies.

## Author summary

Watermelon crops worldwide are threatened by Fusarium wilt, a destructive disease caused by the fungus *Fusarium oxysporum* f. sp. *niveum* (Fon). This pathogen evolves rapidly, overcoming plant defenses and creating new, more aggressive strains. Yet, the molecular mechanisms underlying Fon virulence and evolution remain poorly understood. To address such problems, we assembled and annotated the complete gap-free genomes of three Fon isolates (FonR1, FonR2, FonR3) affiliated with physiological races 1, 2, and 3, respectively. Comparative genomics and transcriptomics of the three isolates revealed unique genetic differences, including accessory chromosomes and isolate-specific effectors, small secreted proteins, which likely contributed to their varying virulence on watermelons. We identified 13 effectors present only in FonR3 isolate but not in FonR1 and 2, one of which (*FonR3SE1*) was proven to promote infection through functional experiments. Additionally, we observed that even shared genes behave differently during infection, influencing disease progression. These findings provide critical insights into how Fon attacks watermelons and how its virulence evolves from a genomic perspective, offering a timely resource and roadmap for developing disease-resistant crops and targeted control strategies.

## Introduction

*Fusarium oxysporum* is a widely spread phytopathogenic fungus causing Fusarium wilt in over 120 plant species [1] and considered among the ten most important fungal plant pathogens [2]. The soilborne fungus invades the plant vascular system with mycelium through roots and causes embolism, leading to plant wilting and eventual death. Despite the broad host range of *F. oxysporum* species complex (FOSC), an individual strain of *F. oxysporum* is host-specific [3], and strains virulent to the same host range are grouped into *formae speciales* (f. sp.). Fusarium wilt of watermelon (*Citrullus lanatus* L.) caused by *F. oxysporum* f. sp. *niveum* (Fon) is often regarded as the most devastating disease of watermelon, one of the most important fruit crops with great nutritional and economic values worldwide [4]. Although several watermelon germplasms or cultivars confer resistance to Fusarium vascular wilt [5], the evolution of Fon quickly overcomes the resistance giving rise to various physiological races. So far, four races (race 0, 1, 2, and 3) of Fon have been recognized based on pathogenicity test using watermelon differential cultivars [6–9]. Among the four races, Fon race 0 is the least economically important being non-pathogenic to the majority of commercial cultivars [4]. Watermelon cultivars such as Calhoun Gray and Sugarlee are resistant to Fon race 1. However, such resistance was overcome by Fon race 2 and no resistant resource was available to Fon race 2 until the release of cultivar PI-296341-*FR* and PKR6 [5,10,11]. Unfortunately, Fon race 3, according to recent reports (USA and China), can overcome all tested watermelon cultivars, including the highly resistant PI-296341-*FR* and PKR6 [8,9,12]. Thus, Fon race 3 poses a huge risk for global watermelon production, requiring research and quarantine efforts to contain its dissemination.

Comparative and functional genomics is essential to understanding the pathogen evolution and dissecting the molecular mechanisms underlying the fungal virulence. *F. oxysporum* genomes, pathogenic or not, are typically organized into two compartments, the core chromosomes (CCs) and accessory chromosomes (ACs) [3]. CCs are genomically conserved among *F. oxysporum*, gene-rich, and vertically transmitted, whereas ACs are typically repeat-rich, lineage- or strain-specific, and could be horizontally transferred among strains. *F. oxysporum* generally has 11 CCs which encode genes essential to survival and reproduction, whereas the number of ACs is highly variable among different *formae specialis* or even races, and AC genes are associated with the fungal pathogenicity and host specificity of the strain. Furthermore, ACs have distinctive characteristics in genomic compositions than CCs such as gene-sparse, low GC content, high transposable element (TE) density, and H3K27me3 methylation indicative of heterochromatin status [13], presenting criteria to identify the ACs in *F. oxysporum* genomes. Genomic analysis of *F. oxysporum* has shown that ACs are enriched in effectors as virulence factors helping pathogens in colonization and infection of host cells. Effectors are small secreted proteins carrying signal peptides, rich in cysteine residues and induced by host infection [14,15]. After the secretion into plant-pathogen interfaces, effectors can either kill host cells with toxicity or bind with the target proteins of host plants to inhibit their immune responses and manipulate host cell activities [16,17]. Interestingly, effector genes are usually arranged in flexible genomic regions with high TE contents, which might aid the rapid evolution and loss-and-gain of virulence and host specificity [16]. Therefore, effectors are potentially key players in Fon virulence to watermelon and the different repertoire of effectors in Fon races could account for the race evolution. Effector studies in Fon have been limited so far, requiring high-quality reference genomes and annotations. There have been published genome assemblies for different Fon races [18]. However, the published Fon genomes are draft assemblies at the contig-level, containing many gaps, given the highly repetitive nature of *F. oxysporum* genomes, which are notoriously difficult to assemble. Currently, gap-free Fon genomes have not been reported, and there have not yet been in-depth comparative genomics and transcriptomics studies among various races to understand the genetic basis and virulence evolution.

In this study, we assembled and annotated gap-free genomes for three isolates from Fon races 1, 2, and 3, and performed comparative genomics and transcriptomics to identify putative determinants of virulence differentiation in Fon races. Genome comparison identified CCs and ACs from three race isolates and revealed conserved and unique accessory regions and chromosomes. Comparative transcriptomics uncovered different temporal patterns of gene expression in three races, even for orthologous genes, especially related to cell-wall degradation. We further identified FonR3 (from race 3) specific effectors essential for its full virulence on previously resistant cultivar PKR6. Our study provided valuable and high-quality genomic resources as well as effector profiles for studying the virulence mechanism of Fon. The results also shed light on the contribution of isolate-specific effectors on the emergence of new pathogenic races and facilitate breeding resistant watermelon cultivars.

## Results

### Pathogenicity and phylogenetics of three Fon physiological races

**Fig 1:**
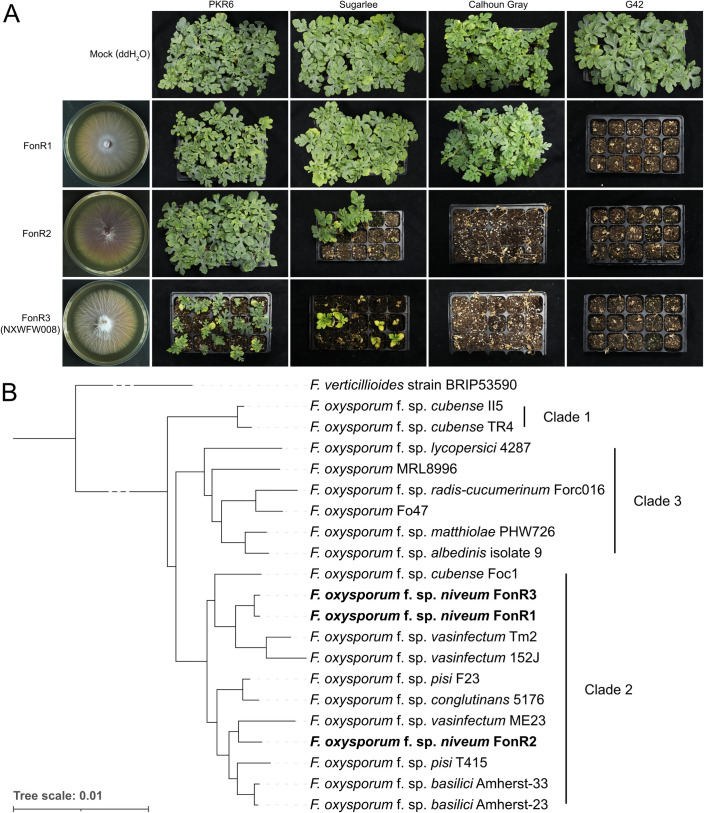
Phenotypes and phylogeny of three isolates of *Fusarium oxysporum* f. sp. *niveum* (Fon) sequenced in this study. **(A)** Potato dextrose agar (PDA) cultures of FonR1, FonR2, and FonR3 (NXWFW008) and disease phenotypes on differential hosts. PDA cultures were photographed 5 days post inoculation (dpi), while the plant infections were photographed 28 dpi on 11-day-old seedlings. Mock was inoculated with water. Different watermelon cultivars used to identify the physiological races of Fon were PKR6 (resistant to R1, R2, but susceptible to R3), Sugarlee and Calhoun Gray (resistant to R1, but susceptible to R2 and R3), and G42 (susceptible to all three races). For each treatment, 15 plants were inoculated, and the experiment was repeated three times. **(B)** Phylogenetic tree of 20 *F. oxysporum* strains including FonR1, FonR2, and FonR3. The tree was rooted by *F. verticillioides* strain BRIP53590. All branches are supported by 100% bootstrap values. Clades were annotated as described in O’Donnell et al. [20].

For this study, we obtained three Fon isolates (Fon-1, Fon-2, and Fon-3) from the Crop Genetics and Breeding Platform of Peking University Institute of Advanced Agricultural Sciences. To verify their pathogenicity to cause Fusarium wilt of watermelons, the three Fon isolates were inoculated against a set of watermelon differential cultivars used in pathogen race identification including G42 (susceptible to Fon race 1, 2, and 3), Calhoun Gray and Sugarlee (susceptible to Fon race 2 and 3 but resistant to race 1), and PKR6 (susceptible to Fon race 3 but resistant to race 1 and 2) [5,8,11,19]. Pyramiding multiple disease resistance, PKR6 is a newly released inbred line conferring high-level resistance to Fon race 2 but not 3, and thus chosen as a key differential host to identify race 3 and investigate the specific effector repertoire of race 3 [11]. Our greenhouse bioassay results showed that Fon-1 was pathogenic only to G42, leading to 100% seedling death of G42 (Fig 1A). Fon-2 overcame G42 and Calhoun Gray completely, and caused 80% death incidence on Sugarlee, but caused no detectable symptoms at PKR6 seedlings (Fig 1A). In contrast, Fon-3 caused vascular wilt symptoms such as stunting, yellowing, wilting or plant death on seedlings of all four tested cultivars (Fig 1A). Therefore, based on the disease symptoms of watermelon differential hosts, Fon-1, Fon-2, and Fon-3 were verified to be Fon race 1, 2, and 3, named FonR1, FonR2 and FonR3, respectively. To understand the evolutionary relationship of the three races with other FOSC members, we constructed a phylogenetic tree including three Fon isolates and 17 other *F. oxysporum* strains rooted with *F. verticillioides* strain BRIP53590 single-copy orthologous genes (see Methods) and annotated the clades as described in O’Donnell et al. [20] (Fig 1B). All three Fon isolates were located in Clade 2, where FonR1 and FonR3 were grouped in one subclade and phylogenetically closer to each other, but both were distant from FonR2, located in another subclade. Two subclades containing Fon isolates also harbored other *formae speciales* (Fig 1B). Reflecting a complex evolutionary route for Fon races, this result was consistent with a previous phylogeny analysis revealing that Fon and other members of FOSC were generally polyphyletic and dispersed in distinct clades or subclades [16]. To understand the genomic basis underlying virulence differentiation, the three isolates belonging to three pathogenic races of Fon were subjected to downstream whole-genome sequencing, assembly, annotation, and comparative analysis.

### Gapless genome assemblies and annotations of isolates belonging to three Fon races

**Fig 2:**
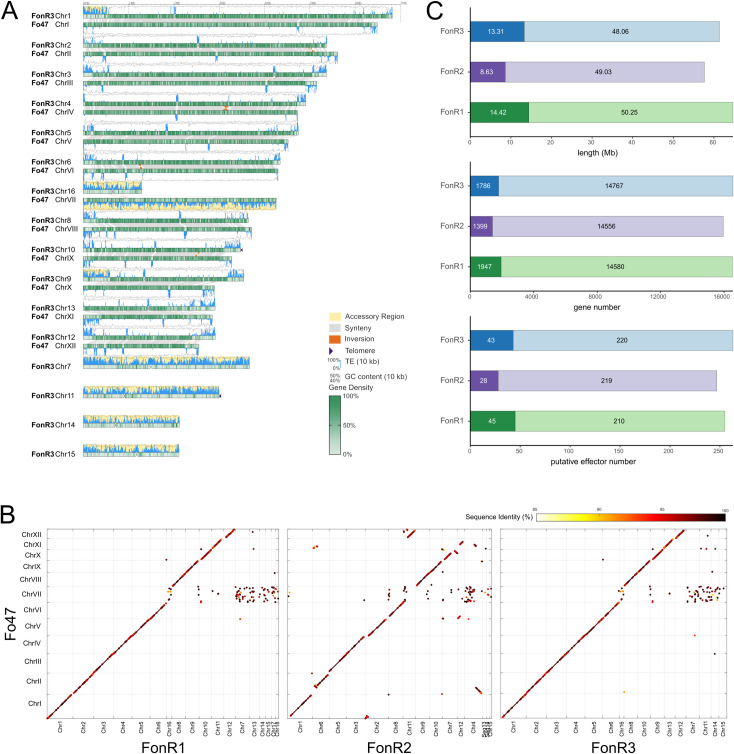
Gap-free genome assembly of FonR3 and accessory genome identification of FonR1, FonR2, FonR3. **(A)** An ideogram of the most virulent race, FonR3, compared to the Fo47 genome was visualized using GenomeSyn [80]. Genomic features, including gene density, GC content, TE content, telomeres, centromeres, and genome synteny with Fo47 chromosomes, are shown on the ideograms. The yellow background indicates the accessory regions. **(B)** Dotplots of FonR1, FonR2, and FonR3 genome alignments with Fo47. The color scale shows the sequence identity of the aligned sequences. **(C)** Comparison of three isolates in terms of total length, the number of annotated genes, and predicted effectors in core (light colors) and accessory (dark colors) genomic regions.

To understand the evolution of pathogenicity in Fon race isolates at the genomic level, we *de novo* assembled high-quality gapless reference genomes of FonR1, FonR2, and FonR3, which will reveal how the three race isolates differ at the chromosome level. We first generated high-coverage sequencing data from each race using a combination of sequencing technologies, including PacBio high-fidelity (HiFi) long reads, Oxford Nanopore Technology (ONT) ultra-long reads, and high-throughput chromatin conformation capture (Hi-C) sequencing reads. Three draft genomes were assembled from HiFi and ONT ultra-long reads using Hifiasm [21], followed by scaffolding to pseudochromosomes using Hi-C reads using the Juicer/3D-DNA pipeline [22,23]. The Hi-C contact maps indicated 18, 13, and 16 pseudochromosomes in the FonR1, FonR2, and FonR3 genome assemblies, respectively (S1 Fig). The ONT reads were applied to fill the remaining gaps in the initial assemblies using TGS-gapcloser [24], obtaining three gapless genome assemblies (S2 Fig). The chromosome IDs of the assemblies were sorted in order of decreasing chromosome length. The assemblies had 64.66, 57.66, and 61.36 Mb genome sizes and contig N50 lengths of 3.72, 4.34, and 4.34 Mb for FonR1, FonR2, and FonR3, respectively (Table 1). All centromeres and 41.5% of the telomeres (20/36, 17/26, and 2/32 in FonR1, FonR2, and FonR3, respectively) were successfully captured (Figs 2A and S3). To validate the assemblies, we mapped raw sequencing reads to each assembly, showing a high mapping rate of HiFi (98.72%-99.79%), ONT (99.82%-100%), and Illumina (92.20%-94.60%) reads. In addition, the Benchmarking Universal Single-Copy Ortholog (BUSCO) assessment [25] resulted in 99.4%, 99.5%, and 99.4% completeness with QV values of 46.4, 57.29, and 40.36 for FonR1, FonR2, and FonR3, respectively (Table 1). Together, these indicate high accuracy and completeness of the three gapless genome assemblies.

**Table 1:** Genome assembly and annotation statistics for isolates belonging to three races of *Fusarium oxysporum* f. sp. *niveum.*

Statistics	FonR1	FonR2	FonR3
Genome size (bp)	64,668,425	57,664,902	61,367,986
Pseudochromosome number	18	13	16
Contigs > 500 kb	18	15	16
Contigs > 50 kb	45	49	19
Contig number	75	82	86
Contig N50 (bp)	3,727,044	4,345,875	4,344,065
Contig N90 (bp)	1,413,554	1,004,243	2,123,745
Gap number	0	0	0
GC content (%)	47.83	48.09	47.67
TE content (%)	20.35	18.51	16.82
BUSCO completeness (%)	99.4	99.5	99.4
QV	46.14	57.29	40.36
Gene number	16,527	15,955	16,553
BUSCO completeness for genes (%)	97.1	99.5	97.5
TE content (%)	20.35	18.51	16.82
TE length (bp)	13,160,780	10,675,578	10,324,453
Retroelements (%)	4.13	3.51	4.23
LTR elements (%)	2.58	2.23	2.56
DNA transposons (%)	6.01	5.7	5.84
Putative effector number	255	247	263

We then annotated the three gapless genomes by integrating *ab initio* prediction, homology, and transcriptome evidence using the MAKER pipeline [26]. To ensure accurate annotation of genes involved in fungal virulence, we sequenced transcriptomes (RNA-seq) of watermelon plants (G42) infected by each of three isolates at 1, 3, and 6 days post inoculation (dpi) using Illumina paired-end sequencing, with fungal mycelia harvested from PDB culture (0 dpi) as control. The Fon genome assemblies were first masked for the repeat sequences identified through RepeatMasker [27], followed by protein-coding gene calling. A total of 16,527, 15,955, and 16,553 protein-coding gene models were predicted for FonR1, FonR2, and FonR3, respectively (Table 1), 36.94%, 37.67%, and 37.23% of which can be functionally annotated by either Gene Ontology (GO) or Kyoto Encyclopedia of Genes and Genomes (KEGG). The comparison of the protein-coding genes using OrthoFinder suggested that 391, 1113, and 464 were specific genes in FonR1, FonR2, and FonR3, respectively. The repeat contents detected by RepeatMasker in whole genomes for FonR1, FonR2 and FonR3 were 20.35%, 18.51%, and 16.82% where the retroelement contents were 4.13%, 3.51%, and 4.23%, and the DNA transposon contents were 6.01%, 5.70%, and 5.84%, respectively (Table 1).

### Structure of Fon core and accessory chromosomes/regions

*F. oxysporum* genomes are well known to have two major compartments: core and accessory genomes, with the latter able to horizontally transfer among different strains and thus contribute to the evolution of pathogenicity [3]. Since core chromosomes are shared among *F. oxysporum* strains, to identify core and accessory genomes for the three new assemblies, we performed whole-genome sequence alignment of FonR1, FonR2, and FonR3 assemblies against the complete genome of a nonpathogenic *F. oxysporum* strain Fo47 (NCBI Accession: GCA_013085055.1) [28] which has 11 core and one accessory chromosomes (Figs 2A, 2B and S3). Overall, the 11 core chromosomes of Fo47 were well aligned with 11 chromosomes in each Fon race despite a low alignment rate for repeat-rich regions, indicating that these were Fon core chromosomes (Figs 2A and S3). We then defined the Fon accessory genomes as genomic regions longer than 400 kb, unaligned with Fo47 core chromosomes, and displaying the genomic characteristics of *F. oxysporum* accessory chromosomes, *e.g.,* low gene density and high repeat content. Under such stringent criteria, 14.42, 8.63, and 13.31 Mb sequences representing accessory genomes were identified in FonR1, FonR2, and FonR3, respectively (Fig 2C). Specifically, Chr07, Chr13–18 in FonR1, Chr15 in FonR2, Chr07, Chr11, Chr14–16 in FonR3 were assigned as accessory chromosomes (Figs 2A and S3). In addition, parts of Chr01, Chr10, and Chr11 in FonR1, unmapped segments Chr13 and Chr14 and parts of Chr01, Chr02, Chr04, Chr07, and Chr11 in FonR2, and parts of Chr01, Chr09 in FonR3 were identified as accessory regions. Interestingly, accessory chromosomes or regions in FonR1 and FonR3 were mostly standalone without attaching to other chromosomes, while accessory genomes in FonR2 mainly exist as “accessory/core chimera chromosomes” likely resulted from large-scale genome rearrangement in FonR2 (also visible in Fig 2B). Notably, unlike other accessory regions attached to ends of core chromosomes, a particular accessory region (2.27-3.38 Mb) of FonR2 resided in the middle of the chromosome (Chr07) surrounded by two core regions (S3 Fig), an observation that has not been reported in *F. oxysporum* genomes so far. The core-accessory boundaries were confirmed by both long-read alignments spanning across the junctions and the Hi-C contact maps, ruling out potential mis-assembly for this chimeric chromosome (S4 Fig). Moreover, there were major genomic inversion events inside Chr06 and at each end of Chr07 in FonR2 compared to the Fo47 reference (Fig 2B), whereas FonR1 and FonR3 only had some minor inversion events.

### Comparative genomics reveals isolate-unique accessory chromosomes/regions

**Fig 3:**
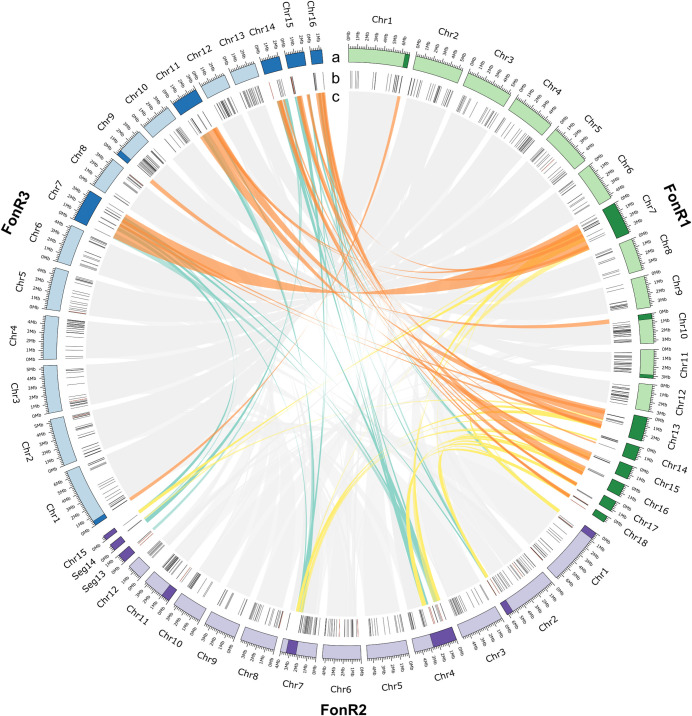
Comparative genomics of FonR1, FonR2, and FonR3 gap-free genome assemblies reveals core and lineage-specific accessory regions/chromosomes. **(a)** Chromosomes of FonR1, FonR2, FonR3. Light colors represent core chromosomes (CC), while dark colors represent accessory chromosomes/regions (AC/AR). **(b)** Predicted effectors (black) and unique effectors (red) are shown. **(c)** Links showing synteny between three genome sequences. Gray links are between CCs while colored links are between AC/ARs.

Given the distinctive virulence profiles of Fon races on differential hosts, their genomes likely harbor unique DNA elements that determine their race-specific virulence traits. To identify such elements, we performed a synteny analysis of the three genomes and found that the three isolates shared a high level of synteny (82.78% synteny on average whole genome) in both core chromosomes and accessory chromosomes (Fig 3). In addition to the 11 conserved core chromosomes (average 84.77% gene synteny among three isolates’ CC), some accessory chromosomes were also syntenic between three isolates. For example, synteny was observed for accessory chromosome Chr07 of FonR1 and FonR3, and the accessory regions on Chr04 in FonR2. GO enrichment analysis showed that transcription regulation-related genes were enriched in these chromosomes and regions in all isolates (S6 Fig). To compare the gene compositions of the three genomes, we performed gene homology analysis on the predicted gene models using OrthoFinder [29]. We identified 16,464 orthogroups across three isolates, among which 11,513 were single-copy orthogroups. There were 391, 1113, and 464 isolate-specific genes in FonR1, FonR2, and FonR3, respectively. 59.1% (231/391), 31.6% (352/1113), and 48.9% (227/464) of isolate-specific genes in FonR1, FonR2, and FonR3 were found in isolate-specific accessory regions (S7 Fig).

**Fig 4:**
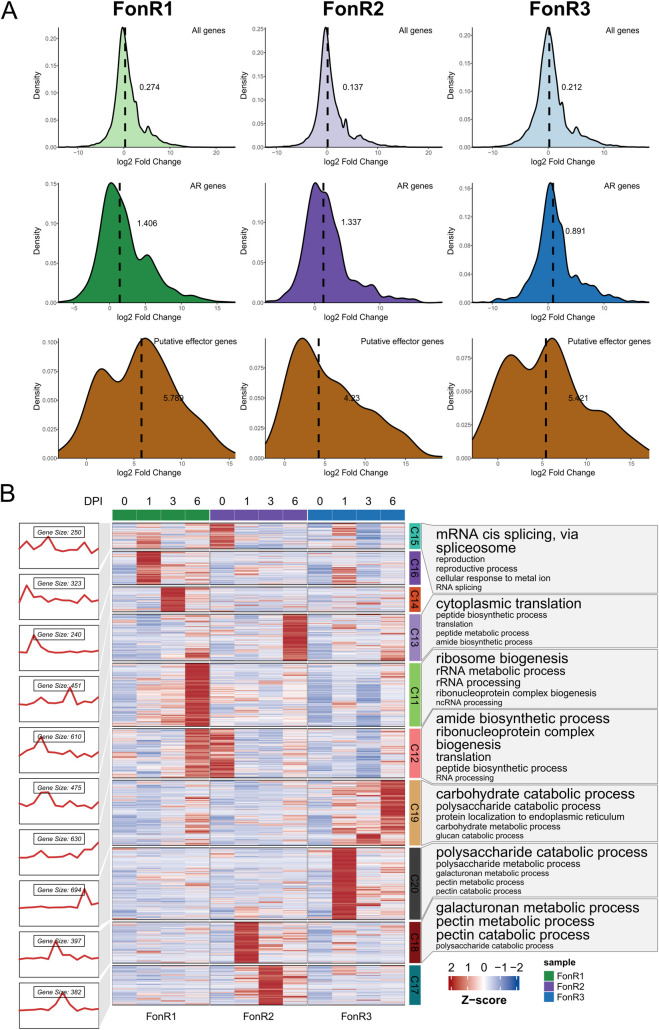
Comparative transcriptomics of three Fon race isolates during watermelon infection. **(A)** Kernel density plots of gene expression log_2_FC(Fold Change) values between control and 6 dpi of the G42 cultivar. The median log_2_FC values are shown as bold numbers and dashed vertical lines. **(B)** k-means clustering of the expression of the single-copy orthologous genes of FonR1, FonR2, and FonR3. The figure was generated using ClusterGVis [72] with k = 20. Only the clusters with a difference among the three isolates are shown. The full image, including all clusters, can be found in S5 Fig.

As accessory genomes are hotspots of virulence genes likely contributing to race evolution in *F. oxysporum*, we investigated the gene expression of core and accessory genomes in FonR1, FonR2, and FonR3 during the infection of watermelon susceptible cultivar G42. While the global gene expression shows little skewness in response to Fon infection (median log_2_FC = 0.137-0.195), the expressions of accessory genes and putative effectors were skewed towards up-regulation by infection (median log_2_FC = 0.891-1.406), suggesting more induced expression of accessory genes (Fig 4A). We then identified differentially expressed genes (DEGs) between the control and 6dpi samples. We observed significantly more infection-stage DEGs from accessory genomic regions than from core ones. For example, 15.36% (1217/7922), 15.19% (1245/8194), and 14.10% (1166/8267) of the genes in core genomes were significantly up-regulated during infection in FonR1, FonR2, and FonR3, respectively. By contrast, 27.27% (177/649), 24.14% (127/526), and 25.50% (166/651) of the genes in accessory genomes were significantly up-regulated during infection (Fig 4A). These observations indicated that a higher portion of genes in accessory regions were up-regulated during infection than the core regions, indicating their roles in Fon infection. Among the single-copy orthologues, 10,006 genes were expressed in at least one condition and only 1944 (16.9%) genes exhibited different expression patterns among the three isolates (S7 Fig), suggesting that the expression of the orthologous genes was mostly similar, despite showing some divergence.

### Transcriptome clustering reveals conserved and divergent gene expression modules

To detect the differences in expression patterns in the conserved genes shared by FonR1, FonR2, and FonR3, we performed k-means clustering (k = 20) on the TPM expression matrix of the expressed single-copy orthologs (SCOs) (Figs 4B and S8). According to the results, the genes in Cluster 20 (C20) and C19 are up-regulated only in the FonR3 post-inoculation samples. Particularly, SCOs in C20 exhibited expression patterns of earlier and higher up-regulation in planta in FonR3 and were enriched with GO terms including “polysaccharide metabolic process”, “galacturonan metabolic process”, and “pectin metabolic process”, suggesting their involvement in plant cell wall degradation. In addition, C20 contained an SCO encoding a pectate lyase C “FonR3Chr10G008700” reported as a plant virulence factor previously [30]. C19 with SCOs enriched in “carbohydrate catabolic process” showed higher upregulation in FonR3 than FonR1 and FonR2. Significantly, three SCOs “FonR3Chr12G002170”, “FonR3Chr09G004770”, and “FonR3Chr01G010130” encoding glycosyl hydrolases 5, 6, and 7, respectively, exhibited a 768–1120-fold expression increase in FonR3 than in FonR1 and FonR2 (65–177-fold change) during infection (Fig 4B). The different expression patterns of SCOs across three isolates indicated that FonR3 might be equipped with different transcription regulators than FonR1 and FonR2 during infection. Furthermore, to study the expression patterns of genes unique in each race, we performed separate k-means clustering (k = 20) for FonR1, FonR2, and FonR3 using their gene expression matrices, followed by functional enrichment analysis. Interestingly, the GO term “polysaccharide metabolic process” indicative of plant cell degradation was enriched in clusters of all three isolates but was present in clusters with different expression patterns (C18 in FonR1, C3 in FonR2, and C17 in FonR3 in S9–S11 Figs). For example, for FonR1 and FonR2, genes in the “polysaccharide metabolic process” enriched clusters were up-regulated at 3 and 6 dpi. However, in FonR3, this up-regulation began at an earlier stage of infection (1 dpi). This indicated that the polysaccharide metabolic process regulation might play a part during infection and the differences in the gene expression patterns of this process might be associated with the differences in virulence.

### Effectorome analysis reveals putative isolate-specific effectors

Effectors, defined as small secreted proteins, are crucial to the successful infection of plant hosts for *F. oxysporum* [31,32]. Considering the importance of effectors, we comprehensively predicted the putative fungal effectors in three Fon race isolates using a pipeline involving three tools commonly used in fungal secretome and effector identification: EffectorP [33], SignalP [34], and TMHMM [35]. The candidate effectors were identified by overlapping EffectorP and SignalP results and removing TMHMM hits, resulting in 467–489 candidate effectors in three isolates (S5 Fig and S1–S3 Tables). Furthermore, we screened out the putative effectors that were highly expressed in post-inoculation samples and little or no expression in control samples (fungal cultures) based on expression patterns derived from hierarchical clustering of transcriptome data, identifying 255, 247, and 263 putative effectors in FonR1, FonR2, and FonR3, respectively, located both on core and accessory chromosomes (Fig 2C). Captured in the putative effectors of three Fon race isolates was *FonSIX6* (FonR1Chr18G000080, FonR2Chr08G000200, FonR3Chr15G000080), which was experimentally demonstrated as an avirulence gene in Fon-watermelon pathosystem [36,37]. These results suggested the robustness of predicted putative effectors. K-means clustering of SCOs suggested these putative effectors are distinctively regulated in three race isolates during watermelon infection, where 67 of 240 single-copy effectors belonging to two clusters (C19 and C20) were only upregulated in FonR3 during early infection.

So far, no resistant watermelon germplasm resources or commercial cultivars to the Fon race 3 have been reported [8], therefore Fon race 3 poses a potential and serious threat to future watermelon production worldwide despite its sporadic reports. Given the importance of Fon race 3, our study focused on identifying and dissecting its virulence factors, particularly functional effectors that enabled Fon race 3 to overcome the resistance in cultivar PI296341 and PKR6. Comparative genomic analyses showed that 11, 10, and 13 putative effectors were unique to FonR1, FonR2, and FonR3, respectively (S5 Fig). We conducted an in-depth analysis of the 13 FonR3-specific effectors (FonR3SEs) that were only present in FonR3 compared to FonR1 and FonR2. It is noteworthy that ten of the thirteen FonR3SEs are located on core chromosomes and three on accessory regions, indicative of both vertical and horizontal inheritance of FonR3SEs (Fig 3). Transcriptome data showed that all 13 *FonR3SE* genes were expressed at much lower levels during the vegetative growth stage but highly expressed *in planta* (S3 Table and S12A Fig). Moreover, most mature proteins of 13 FonR3SEs had fewer than 224 amino acid residues, were rich in cysteines, and possessed N-terminal signal peptides, well aligned with characteristics of effectors (S4 Table).

### FonR3SE1 is a key virulence factor for FonR3 infection of watermelon

To study the functions of putative FonR3SEs, we generated the gene deletion mutants via the gene replacement approach in wild-type (WT) FonR3 and obtained eight of thirteen FonR3SE mutants (S4 and S5 Tables). In a preliminary greenhouse bioassay on watermelon cultivar PKR6, si**x** of eight candidate *FonR3SE* deletion mutants (Δ*FonR3Chr02G017230*, Δ*FonR3Chr10G008340*, Δ*FonR3Chr01G001850*, Δ*FonR3Chr09G010100*, Δ*FonR3Chr15G000140*, Δ*FonR3Chr15G000180*) exhibited significant increase in fresh weight of infected seedlings compared with WT FonR3 (S12B Fig), and among them three deletion mutants (Δ*FonR3Chr02G017230*, Δ*FonR3Chr10G008340*, Δ*FonR3Chr09G010100*) also significantly reduced virulence by causing weakened disease index (S12C Fig). Although the isolate FonR3 probably employs a group of FonR3-specific effectors to overcome the previously resistant cultivar PKR6, Δ*FonR3Chr02G017230* (Δ*FonR3SE1*) appeared to be the least virulent on PKR6 (S12B, S12C Fig) and therefore was subject to downstream functional analysis.

**Fig 5:**
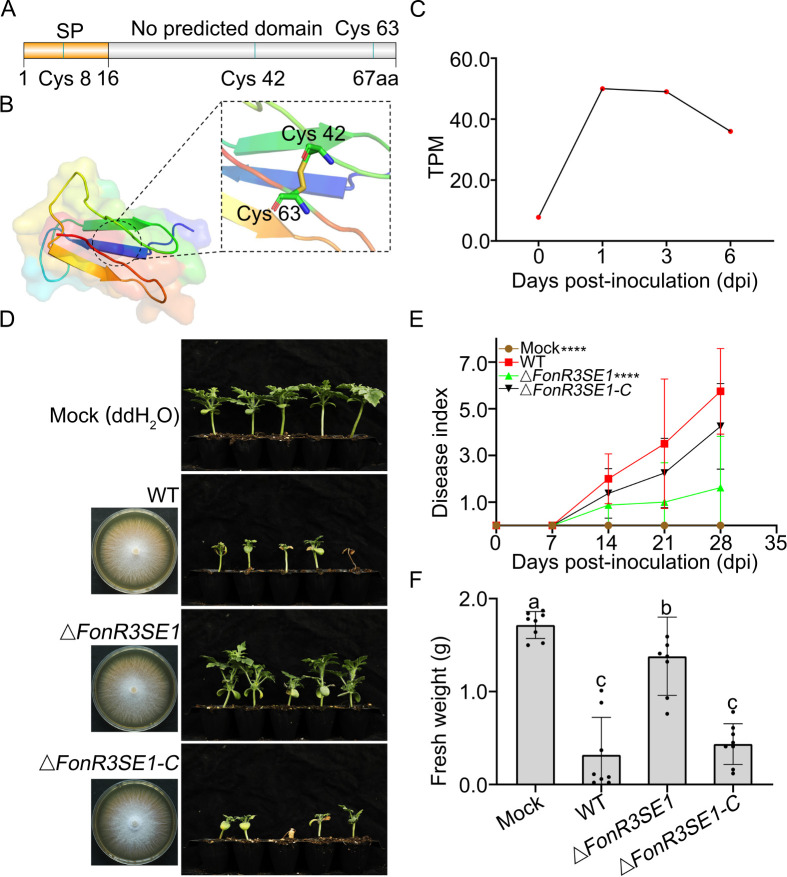
Features of FonR3SE1, a critical effector for FonR3 infection. **(A)** A structure diagram for the *FonR3SE1* gene with domains and cysteine residues. **(B)** Predicted 3D protein structure of FonR3SE1 without signal peptide using Alphafold2 and ColabFold software [40,81]. **(C)** The expression of *FonR3SE1* in the vegetative growth stage (0 dpi) and *in planta* (1, 3, 6 dpi). **(D)** Greenhouse bioassays for the functional study of *FonR3SE1* were repeated independently three times, with eight to ten plants for each treatment. One representative experiment was shown here. Left lane: colony of WT FonR3, the *FonR3SE1* mutant, and its complemented strain on PDA plates at 5 dpi; Right lane: Infected PKR6 plants with mock (water) and the corresponding strains from the left at 28 dpi using 11-day-old seedlings. **(E)** Disease index quantification of infected PKR6 seedlings from 7 to 28 dpi. Disease index was evaluated based on a 7-scale rating: 0 = asymptomatic, 1 = slight stunted growth and yellowing, 3 = stunted growth and yellowing, 5 = wilting, 7 = dead. Wilcoxon rank-sum test between 28 dpi disease indices of WT and mutants was applied. *p*-value < 10^-4^ is denoted as ****. **(F)** Fresh weights of the above-ground infected plants at 28 dpi. Different letters above the bars represented significant differences between treatments using ANOVA analysis followed by Duncan’s multiple range test (*p* = 0.05).

The gene *FonR3SE1* is located in core chromosome Chr02 of FonR3 and lacks homologs in FonR1 and FonR2 (S12D Fig). Nonetheless, *FonR3SE1* shares 100% identity with a hypothetical protein (accession KAF6523144.1) of *F. oxysporum* f. sp. *conglutinans* (Foc) strain Fo5176 [38], 97% identity with an uncharacterized protein (KAJ0153318.1) of *F. oxysporum* f. sp. *albedinis* (Foa) and a few other *F. oxysporum* strains. With only 67 amino acids, FonR3SE1 is a small protein consisting of a signal peptide and an unknown domain, aligned with the non-conservative nature of functional domains of effectors [39] (Fig 5A). The predicted 3D structure of FonR3SE1 (excluding the signal peptide) by AlphaFold2-based ColabFold open-source software [40] had an *α*-helix, three *β*-sheets, and several flexible-loop regions (Fig 5B). Cysteine residues of fungal effectors could form multiple disulfide bonds to promote the proper protein folding and stability [41]. FonR3SE1 carries three cysteine residues, which were predicted to form one disulfide bond between cysteine 42 and cysteine 63 (Fig 5B). While sequence homology did not return any similarities to known proteins, according to structure homology, FonR3SE1 shows high similarity to Beta/gamma crystallin proteins (probability: 0.99, e-value: 1.07) (S12E Fig) [42]. The transcription of *FonR3SE1* was maintained at a fairly low level during vegetative growth (0 dpi) but was significantly upregulated after infection was initiated (1, 3, 6 dpi) (Fig 5C and S3 Table).

To further validate the function of *FonR3SE1,* we generated a complemented strain Δ*FonR3SE1*-*C* for Δ*FonR3SE1* (S12F, S12G Fig). The mutant and complemented strain showed no significant differences with WT FonR3 on growth rates, colony morphology, and conidiation, implicating that the absence of *FonR3SE1* had no significant interference with fungal vegetative growth (Figs 5D and S13). The greenhouse bioassays of Δ*FonR3SE1* and Δ*FonR3SE1*-*C* were repeated three times, with eight to ten plants per treatment (Figs 5D, S14 and S6 Table). Besides occasional quick death around 7 dpi, the majority of inoculated PKR6 plants by WT FonR3 started to show leaf yellowing and stunted growth after 7 dpi, and the symptoms continued to develop for 28–56 dpi until complete plant death. In one representative experiment, PKR6 plants infected by WT FonR3 weighed 0.32 g while mock plants weighed 1.72 g on average at 28 dpi (Fig 5E, 5F). The deletion of *FonR3SE1* led to a strong alleviation of the disease index with a significantly increased average fresh weight of 1.38 g of PKR6 seedlings at 28 dpi. The complemented strain Δ*FonR3SE1-C* provoked a level of disease index comparable to WT with an average fresh weight of 0.44 g, indicating a restoration of virulence. Therefore, we inferred that FonR3SE1 was an important virulence effector for the FonR3 to overcome the previously resistant cultivar PKR6*.*

## Discussion

The asexual filamentous fungus *F. oxysporum* is the causal agent of vascular wilt in over 100 different crop species, a major threat to agricultural productivity with few effective control measures. Genomics is the key to understanding the mechanisms behind the pathobiology, especially the rapid evolution of pathogen virulence. Despite their small genomes, it is challenging to fully assemble their genomes due to repeat-rich chromosomes. The genomes of multiple members in FOSC have been reported since the first release of the tomato pathogen *F. oxysporum* f. sp. *lycopersici* (Fol4287) [3], revealing their characteristic two-speed genome structures with horizontally transferred accessory chromosomes. Little is known about the host-pathogen mechanisms of Fon, the causal agent of watermelon vascular wilt of watermelon, due to the lack of high-quality chromosome-level reference genomes. While the genome of different races of Fon was assembled previously [18], these releases are contig-level and fragmented, unable to reveal the chromosome structure and evolution via comparative genomics. Here, we combined the cutting-edge long-read (PacBio and ONT) sequencing and Hi-C sequencing to assemble and annotate the gap-free genomes for three isolates of Fon affiliated with three physiological races showing distinctive virulence towards different hosts. All assembled chromosomes were gapless with all centromeres and most telomeres captured, except for FonR2, which missed 17 telomeres. Before this study, only two gap-free genome assemblies were released for *F. oxysporum*, the endophyte Fo47 [28] and *Arabidopsis* pathogen Fo5176 [43]. Therefore, we expect that the three gap-free Fon genomes from this study will be a great addition to the high-quality genome resources of FOSC to study their pathobiology and genome evolution.

**Fig 6:**
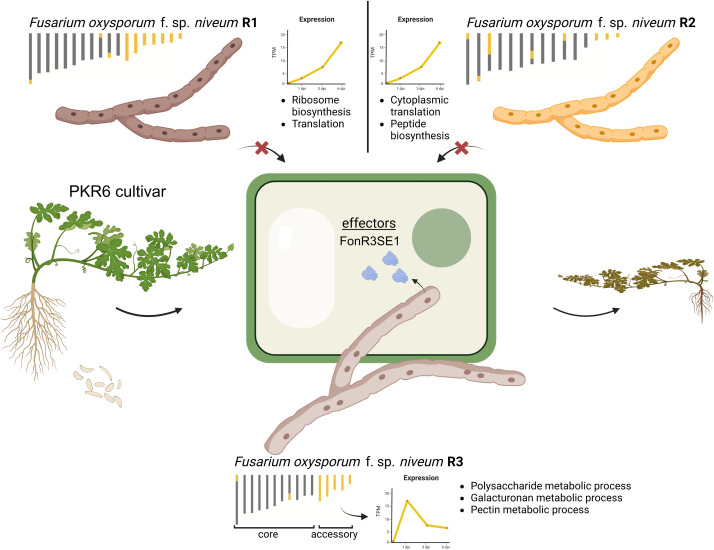
A schematic diagram summarizing the evolution of virulence in *F. oxysporum* f. sp. *niveum* (Fon). Core (gray) and accessory (yellow) chromosomes/regions of FonR1, FonR2, and FonR3, and the accessory genome expression patterns with the enriched GO terms in infection on the susceptible G42 cultivar are shown. While FonR1 and FonR2 cannot infect the resistant cultivar PKR6, FonR3 can infect it and cause wilt symptoms through the FonR3SE1 effector. This image was created by Biorender under an Academic Individual License.

Genome variation is a major driving force for evolution and the generation of new traits. Rapid evolution is a hallmark of fungal pathogens, especially *F. oxysporum,* allowing them to adapt to new environments and resistant cultivars. The different races, especially race 3, of Fon have overcome all known Fusarium wilt-resistant watermelon germlines. Our analysis of the three gap-free genomes yielded a couple of surprising findings. First, we are surprised by the observation of a large difference in their genome structure, such as the number of chromosomes, even at the physiological race level within the same *F. oxysporum* formae speciales. Second, it is also surprising to see the higher sequence similarity and collinearity between race 1 and race 3 than each with race 2, highlighting the uncanonical and complex evolutionary trajectories of the Fon genome and race evolution. Such karyotype differences and the polyphyletic nature of Fon races hinted at potential independent origin and evolution of fungal virulence on watermelon hosts, likely involving a complex history of genetic introgression and recombination. By comparing the gap-free genomes, we dissected the conserved and distinctive genome characteristics behind race evolution, and identified the accessory chromosomes and regions in all three isolates, which showed substantial synteny with high sequence identity, but also had unique accessory genomic regions. Comparison of annotated genes revealed largely conserved gene families with similar expression patterns, reflecting the core gene regulatory machinery for Fon. However, divergence of expression was also observed for conserved genes with and without interaction with host plants, suggesting an evolution of cis- or trans-regulatory elements for these highly conserved coding regions. For example, K-means clustering of single-copy ortholog expression profiles revealed gene modules (C19 and C20) up-regulated exclusively in FonR3 post-inoculation (Fig 6). The fact that most of these genes located on core genomic regions also indicated that genome and transcriptome variation in core regions also very likely contribute to distinctive virulence of races on hosts, although experiments are needed to study these orthologs with diverged expression in future studies of pathogen evolution, which can focus on mapping the regulatory elements and identifying functional genomic variants using population genomic data.

The putative effector profiles of FonR1 (255), FonR2 (247), and FonR3 (263) predicted in this study provided valuable resources for further functional study of Fon effectors. As more high-quality genome assemblies of Fon races from different geographic locations and their putative effector profiles become available, the vital effectors determining host specificity may be further narrowed down. The identification of 13 FonR3SEs in FonR3 highlights critical factors contributing to its pathogenicity on a broad spectrum of watermelon differential cultivars. The mutagenesis experiment of the *FonR3SE1* gene in a core chromosome confirmed its critical role for FonR3 to overcome previously resistant cultivar PKR6 (Figs 5 and 6). Though absent in FonR1 and FonR2, *FonR3SE1* is present in other ff. spp. such as Foc and Foa. Given the polyphyletic nature of Fon and FOSC in general, and the fact that strains from different ff. spp. could be found genetically closer than strains of the same f. sp. [3,16,44], it is not surprising that effectors can be shared between ff. spp. throughout the FOSC vertically and horizontally, giving rise to new pathogenic races

As the genome composition of FOSC is highly diverse and polyphyletic, it is likely that each Fon race, identified using host differentials, might represent a population of fungal strains with similar genetic makeup. Isolates belonging to the same race but from different geographic regions may differ greatly in their genome compositions, such as chromosome numbers or effector repertoire. As a result, the limitation of this study is that one gap-free genome assembly may not capture all genetic variations of its designated race. A thorough understanding of Fon race evolution would be further advanced by the availability of Fon population or pan-genomics using high-quality genomes in the future.

In summary, the three gap-free genome assemblies, annotations, and predicted putative effectors of Fon physiological races are valuable genetic resources for dissecting *F. oxysporum* pathobiology on watermelon, studying fungal genome evolution, and improving the development of effective disease control strategies.

## Materials and methods

### Fungal isolates and plant materials

Three Fon isolates (FonR1, FonR2, FonR3) and seeds of watermelon cultivars (G42, Calhoun Gray, Sugarlee, and PKR6) were kindly provided by the Crop Genetics and Breeding Platform of Peking University Institute of Advanced Agricultural Sciences, Weifang, Shandong. FonR3 (NXWFW008) was recently isolated from infected watermelon plants in Ningxia Province, China [9]. Fungal isolates, including derived mutants generated in this study (S1 Table), were routinely cultured on potato dextrose agar (PDA) plates at 26°C. Conidiation was measured with 5-day-old potato dextrose broth (PDB) cultures, and conidial germination was assayed with liquid yeast extract peptone dextrose (YEPD) broth cultures (0.3% yeast extract, 1% peptone, and 2% dextrose) at 26°C in a rotary shaker at 175 rpm for 12 h [45]. For ultra-long genomic DNA isolation, the vegetative hyphae were harvested from 2-day-old PDB cultures set at 175 rpm and extracted using a cetyltrimethylammonium bromide (CTAB) method [46].

### Greenhouse bioassays

Seeds of watermelon cultivars were surface sterilized in 0.1% mancozeb for 30 minutes before rinsing with distilled water. To improve the germination rate, disinfected seeds were incubated at 30°C for 48 h [10]. Then germinated seeds were sown in perlite and placed in a growth room set at 26°C/16h and 18°C/8h with a humidity of 80%. After 11 days, seedlings were uprooted and washed off perlite. For each treatment, 10 seedlings were inoculated by dipping roots into a spore suspension of 5 × 10^6^ spores/ml for one minute [10,45] and planted in sterilized soil (substrate/perlite/vermiculite = 3:1:1). Mock plants were dipped into water. Inoculated plants were grown in the above growth condition for 28 days. The preliminary greenhouse bioassay was conducted once and the functional validation of *FonR3SE1* was conducted three times independently. Disease phenotypes were monitored and documented once a week according to a disease index scale: 0 = asymptomatic, 1 = slight stunted growth and yellowing, 3 = stunted growth and yellowing, 5 = wilting, and 7 = dead. The Wilcoxon rank-sum test was used to evaluate the change in disease index with respect to the WT at 28 or 35 dpi. Plant tissues above the soil were harvested and weighed at 28 dpi or 35 dpi. One-way ANOVA with Duncan’s multiple range test was performed for the fresh weight of the plants.

### Genome and transcriptome sequencing

Illumina paired-end DNA sequencing library was prepared using NEB Next Ultra DNA Library Prep Kit for Illumina (NEW ENGLAND BioLabs) following the manufacturer’s instructions. The 150 bp paired-end reads were produced using the Illumina Novoseq 6000 platform by Novogene Biotechnologies Inc. (Tianjin, China). PacBio SMRTbell library was constructed using PacBio SMRTbell Express Template Prep Kit 2.0 (PacBio). Short reads were removed with a 15 kb cutoff on BluePippin (Sage Science). HiFi consensus reads were generated using a PacBio Sequel II system at Novogene Biotechnologies Inc. (Tianjin, China). The Nanopore DNA library was prepared following the Ligation Sequencing SQK-LSK109 Kit (Oxford Nanopore Technologies, Oxford, UK) protocol and sequenced using the Oxford Nanopore GridION (20 kb) platform. The Hi-C library was prepared from cross-linked chromatins with a standard Hi-C protocol [47]. Then the library was sequenced using Illumina NovaSeq 6000 to obtain 150 bp paired-end reads at Novogene Biotechnologies Inc. (Tianjin, China). Total RNA was extracted using Trizol Reagent (Thermo Fisher Scientific). The mRNA was subjected to transcriptome sequencing library construction using the Illumina TruSeq transcriptome kit (Illumina). The libraries were then sequenced using the Illumina Novaseq 6000 platform at Biomarker Technologies Corporation (Qingdao, China) to generate 150 bp paired-end reads.

### Genome assembly

Genome sizes of the three isolates were estimated using the Illumina data by Jellyfish v2.3.0 [48] (k-mer size = 19) and Genomescope v1.0 [49]. HiFi and ONT reads were assembled with Hifiasm v0.19.5 [21]. To remove within-species contamination and acquire the nuclear genome, we aligned the contigs of the initial assembly to the reference genome of *Fusarium oxysporum* mitochondria (GenBank accession: NC_017930) with Minimap2 v2.24 [50]. Contigs with above 50% coverage were removed. To remove bacterial contamination, we ran a megaBLAST [51] search against a database of common contaminants in eukaryotic genome assemblies (ftp://ftp.ncbi.nlm.nih.gov/pub/kitts/contam_in_euks.fa.gz) and the reference genome of the contamination bacteria (*Brucella intermedia*, GenBank accession: GCA_900454225.1) we detected in FonR2 samples. Then we remove the contamination contigs with the following criteria [52]: requiring e-value ≤ 1 × 10^−4^, reporting matches with ≥ 98% sequence identity and match length 50–99 bp, ≥ 94% and match length 100–199 bp, or ≥ 90% and match length 200 bp or above. To anchor the contigs to chromosomes, we applied the pipeline of Juicer v1.5 [22], 3D-DNA v180419 [53], and Juicebox v1.11.08 [23]. The anchored scaffolds were manually examined and adjusted within Juicebox for assembly validation. To assess genome completeness, we applied BUSCO v5.4.3 [25] for ortholog detection using fungi_odb10 database. Quality values (QV) based on HiFi reads were estimated using Merqury v1.3 [54] with the k value of 23.

### Genome annotation

To annotate repeats in the genome assemblies, we used the universal Repbase database and a species-specific de novo repeat library constructed by RepeatModeler v2.0.6 [55] to annotate the DNA sequences and then annotated and masked the repetitive elements by RepeatMasker v4.1.2 [27]. To predict gene models, we assembled the RNA-Seq reads with HISAT2 v2.1.0 [56] and Stringtie v2.2.1 [57] for transcriptome evidence and downloaded the protein sequences of *Fusarium oxysporum* and the universal fungi protein knowledgebase from Swiss-Prot [58] for homologous protein evidence. Transcriptome-based prediction, protein homology-based prediction, and *ab initio* prediction were combined in the pipeline of MAKER v3.01.03 [26] and BRAKER v2.1.6 [59]. We first trained the GeneMark-ET and AUGUSTUS models using BRAKER and trained the SNAP semi-HMM model using MAKER. Then we integrated the trained SNAP, GeneMark-ET, and AUGUSTUS models into MAKER to predict more credible genes. Finally, the highest-ranking gene sets were retained based on Annotation Edit Distance (AED < 0.5) [60]. Gene functional annotation was conducted using eggNOG-mapper v2.1.12 [61].

### Fungal effector prediction

To select the putative fungal pathogenic effector proteins from the predicted gene models, we first used the neural network of SignalP v6.0 [34]. The predicted signal peptides were examined with TMHMM v2.0 [35] and the peptides with detected transmembrane helices were excluded from the predicted secretome dataset. We then predicted the effector proteins from the putative secretomes using EffectorP v3.0 [33]. To further screen the putative effectors, quantifications of gene transcripts from RNA-seq reads of axenic and post-inoculation samples were performed using kallisto v0.48.0 [62]. The putative effectors with higher expression levels in post-infection samples were selected using the hierarchical clustering in R base functions for experimental verification.

### Gene homology and synteny analysis

Orthologues and orthogroups were inferred using OrthoFinder v2.5.4 [29] with default value settings and ‘-M msa’ activated. We identified the genes in isolate-specific orthogroups as isolate-specific or isolate-unique genes. The phylogenetic tree was generated using the species tree by OrthoFinder using 18 publicly available *Fusarium* genomes and drawn using iTOL v5 [63]. The gene synteny analysis was performed by JCVI v1.1.19 [64]. The syntenic gene blocks were identified by performing an all-against-all LAST search and chaining the hits with a distance cutoff of 20 genes and a minimum block size of 5 gene pairs. Microsynteny analysis was also performed by JCVI with options “-m jcvi.compara.synteny mcscan” and “-m jcvi.graphics.synteny”.

### Transcriptome analysis

Raw transcriptome data were pre-processed with fastp v0.23.2 [65] to trim artifacts and improve data quality. Gene expression levels were then quantified using kallisto v0.48.0 [62]. Counts for mapped reads were normalized by TPM (transcripts per million). To visualize the expression patterns of the focused genes among samples, heatmaps were generated using the R package ComplexHeatmap v2.12.1 [66]. Gene function and protein homology term enrichment analyses based on gene ontology (GO) [67,68] and PFAM [69] databases were performed using ClusterProfiler v4.8.3 [70]. The differential expression analysis was performed using the R package DESeq2 v1.40.2 [71]. We identified genes with log2 fold changes greater than or equal to 2 and adjusted p-values less than 0.05 as significantly up-regulated genes, and genes with log2 fold changes less than or equal to -2 and adjusted p-values less than 0.05 as significantly down-regulated genes. The k-means clustering of gene expression patterns was conducted and visualized using ClusterGVis v0.1.1 [72].

### Generation of knock-out mutants for FonR3-specific effectors

Protoplast preparation and polyethylene glycol (PEG)-mediated transformation were performed as described previously [73]. Briefly, hyphae of FonR3 grown in YEPD for 12 h were lysed with an enzyme lysis buffer containing 25 mg/ml driselase (Sigma-Aldrich, St. Louis, MO, USA) and 5 mg/ml lysing enzymes (Sigma-Aldrich, St. Louis, MO, USA) under a condition of 31°C, 110 rpm for 2–3 h. Then released protoplasts were collected by centrifugation at 3,500 rpm at 4°C [45]. Protoplasts were subsequently washed twice with STC buffer (20% sucrose, 50 mM CaCl_2_, 10 mM Tris pH 7.5) and resuspended to a final concentration of 10^5^ - 10^6^ per ml with STC buffer.

Gene replacement constructs of specific effectors in the FonR3 isolate were generated using a split-marker approach [74]. For each effector gene, approximately 1.0 kb 5’ upstream and 3’ downstream regions were respectively amplified from FonR3 with gene-specific primer pairs 1F/2R and 3F/4R (S2 Table). The resulting PCR products were ligated to the 5’ and 3’ regions of the hygromycin phosphotransferase (*hph*) cassette by overlapping PCR [75,76].

For transformation, 200 μl protoplasts and 5 μg fused constructs were mixed and incubated at 26°C for 20 min, followed by supplementing 1 ml PSTC (40% PEG-8000 in STC buffer) and another incubation of 20 min. Then 10 ml regeneration medium (0.3% yeast extract, 20% sucrose, 0.3% casein enzymatic hydrolysate) was added to the mixture before an overnight incubation in a shaker set at 110 rpm and 26°C. The resulting transformants were selected on Top agar medium (0.3% yeast extract, 0.3% casamino acids, 20% sucrose, and 1.5% agar) with 300 μg/ml hygromycin B (CalBiochem, La Jolla, CA, USA). Positive transformants were identified by PCR using corresponding primers (S2 Table).

### Generation of the complemented strain

For complementation of the deletion mutant Δ*FonR3SE1*, a strong promoter from FonR3 (promoter of FonR3Chr10G008340), and the *FonR3SE1* gene with its 1.5 kb downstream region were amplified with specific primers (S2 Table) and ligated by overlapped PCR. The ligated fragments were cloned into XhoI-digested pFL2 vector by the yeast gap repair approach using yeast strain XK1–25 [77,78]. The recombined constructs carrying the geneticin-resistant marker were rescued from Trp^+^ transformants and transformed into the protoplasts of the mutant. Transformants containing the complementation constructs were screened with geneticin and identified by PCR assays.

## Supporting information

S1 Fig Hi-C contact maps of FonR1, FonR2, and FonR3 genome assemblies. (TIFF)

S2 Fig Confirmation of gap closing in FonR2 assembly. On the left, the chromosomes with gaps shown in black squares in the Hi-C contact maps, and on the right, IGV screenshots of HiFi and ONT read mappings at the gap region.
(TIFF)

S3 Fig Genome alignment of FonR1, FonR2, and Fo47. Ideograms of FonR1 **(A)** and FonR2 **(B)** were visualized using GenomeSyn [80]. Genomic features, including gene density, GC content, TE content, telomeres, centromeres, and genome synteny with Fo47 chromosomes are shown on the ideograms. The yellow background indicates the accessory regions.
(TIFF)

S4 Fig Confirmation of AR/CC border in FonR2. Hi-C contact map showing Chr07 of FonR2 on the left and IGV screenshots with HiFi and ONT reads mappings on the AR/CC borders. AR: accessory regions. CC: core chromosome.
(TIFF)

S5 Fig Fon effector prediction pipeline. The effectors are first identified if they are predicted by SignalP [34] and EffectorP [33] but not including the transmembrane domain predicted by TMHMM [35]. After filtering, only the expressed genes in any of the selected conditions for transcriptome analysis, only genes that have increased expression *in planta* have been selected as putative effectors. Finally, isolate-specific effectors were identified with OrthoFinder [29].
(TIFF)

S6 Fig Gene ontology (GO) enrichment analysis of accessory chromosomes (AC) of FonR1 (left), FonR2 (middle), and FonR3 (right). The color of bubbles is scaled by the adjusted p-values from enrichment analysis. The size of bubbles is scaled by the number of genes annotated with the specific GO terms. BP: biological process. CC: cellular component. MF: molecular function.
(TIFF)

S7 Fig Donut plot summarizing the ratio of the ortholog gene family composition and gene expression conservation in annotated genes of three Fon isolates. (TIFF)

S8 Fig Heatmap showing the transcriptome clustering using K-means (k = 20) clustering of single-copy ortholog expression of three race isolates (FonR1, FonR2, and FonR3) at fungal culture and during infection of watermelon cultivar G42 at different time points (1, 3, and 6 dpi). Significantly enriched gene ontology terms are listed on the right side.
(TIFF)

S9 Fig Heatmap showing the transcriptome clustering using K-means (k = 20) clustering of FonR1 expression at fungal culture and during infection of watermelon cultivar G42 at different time points (1, 3, and 6 dpi). Significantly enriched gene ontology terms are listed on the right side.
(TIFF)

S10 Fig Heatmap showing the transcriptome clustering using K-means (k = 20) clustering of FonR2 expression at fungal culture and during infection of watermelon cultivar G42 at different time points (1, 3, and 6 dpi). Significantly enriched gene ontology terms are listed on the right side.
(TIFF)

S11 Fig Heatmap showing the transcriptome clustering using K-means (k = 20) clustering of FonR3 expression at fungal culture and during infection of watermelon cultivar G42 at different time points (1, 3, and 6 dpi). Significantly enriched gene ontology terms are listed on the right side.
(TIFF)

S12 Fig The virulence profile of eight FonR3-specific effector mutants in causing wilt disease of watermelon seedlings in a preliminary greenhouse bioassay. **(A)** The expression of eight putative FonR3-specific effectors in PDA medium (0 dpi) and during infection on cultivar G42 (1, 3, 6 dpi). **(B, C)** Fresh weights and disease indices of infected PKR6 seedlings by FonR3-specific effector mutants. For each treatment, 10 plants were used. Different letters indicate significant differences based on ANOVA analysis followed by Duncan’s multiple range test (*p <* 0.05). Disease index was evaluated based on a 7-scale rating: 0 = asymptomatic, 1 = slight stunted growth and yellowing, 3 = stunted growth and yellowing, 5 = wilting, 7 = dead. The Wilcoxon rank-sum test was applied to the disease indices between WT and mutants at 35 dpi. *p*-value < 0.01 **, *p*-value < 10^-4^ ****. Note: The *FonR3Chr02G017230* gene was bold and given the name of *FonR3SE1* in this study. **(D)** Microsynteny plot of the *FonR3SE1* loci in FonR1, FonR2, and FonR3. Gray lines show syntenic blocks. Genes on the plus strand are shown in blue, while genes on the minus strand are shown in green. *FonR3SE1* is shown in orange. **(E)** Superposition of FonR3SE1 predicted protein structure (blue) and *Hirsutella minnesotensis* 3608 Beta/gamma crystallin ‘Greek key’ domain containing protein (AF-A0A0F8A4Y3-F1-model_v4, yellow) generated by Foldseek [42]. **(F-G)** PCR detection of the *FonR3SE1* gene in the WT, Δ*FonR3SE1* **(F)**, and Δ*FonR3SE1-C* **(G)**.
(TIFF)

S13 Fig The *FonR3SE1* is dispensable for vegetative growth and conidiation of FonR3. **(A)** Colony diameters of the WT FonR3, Δ*FonR3SE1*, and Δ*FonR3SE1-C* were measured on PDA plates at 25 °C at 5 dpi. **(B)** Conidial production was counted by using a hemocytometer at 3 dpi in PDB medium at 25°C in a 175-rpm shaker. **(C)** Conidial morphology at 3dpi. Scale bar = 50 μm. Mean and standard deviation (SD) of colony diameters and conidiation were calculated from three independent experiments. Different letters indicate significant differences based on one-way ANOVA analysis followed by Duncan’s multiple range test (*p* = 0.05).
(TIFF)

S14 Fig Two more independent biological replicates of greenhouse bioassays for the functional study of *FonR3SE1.* For each treatment, eight to ten plants were tested. **(A-B)** Infected PKR6 plants at 28 dpi using 11-day-old seedlings for inoculations. **(C-D)** The corresponding disease index of infected PKR6 seedlings at 28 dpi. **(E-F)** The corresponding fresh weights of the above-ground infected plants at 28 dpi. Different letters above the bars represent the significant differences between treatments using one-way ANOVA analysis followed by Duncan’s multiple range test (*p* = 0.05).
(TIFF)

S1 Table List of putative FonR1 effector genes and their expression in the watermelon plant. (XLSX)

S2 Table List of putative FonR2 effector genes and their expression in the watermelon plant. (XLSX)

S3 Table List of putative FonR3 effector genes and their expression in the watermelon plant. (XLSX)

S4 Table The information for putative FonR3-specific effectors. (XLSX)

S5 Table The list of primers used in the mutant constructions. (XLSX)

S6 Table The complete data sets for three independent greenhouse bioassays to functionally characterize the putative effector FonR3SE1. (XLSX)
