# Supplementary figures and images for: Gap-free comparative genomics uncover virulence factors for Fusarium wilt of watermelons
Source: PLoS Pathog. 2025 Aug 25;21(8):e1013455. doi: 10.1371/journal.ppat.1013455 (PMC12396751; doi:10.1371/journal.ppat.1013455)

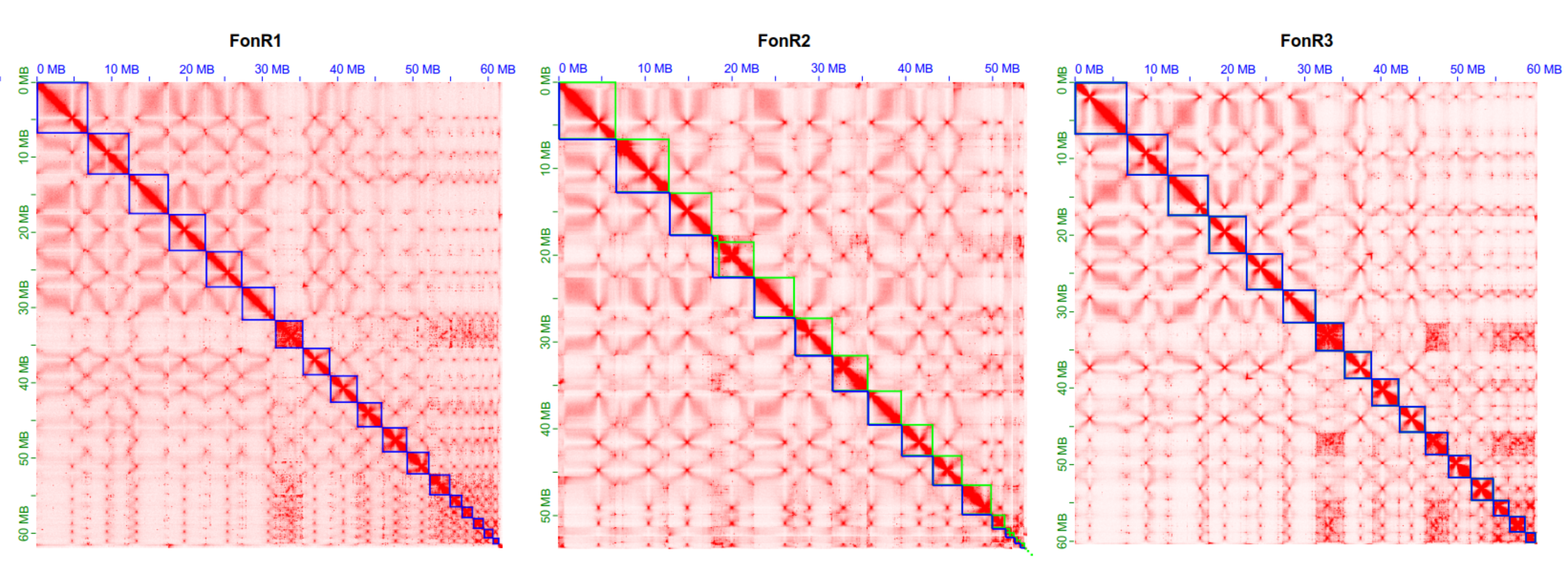

Supplement: S1 Fig — (TIFF) [file ppat.1013455.s001.tiff]

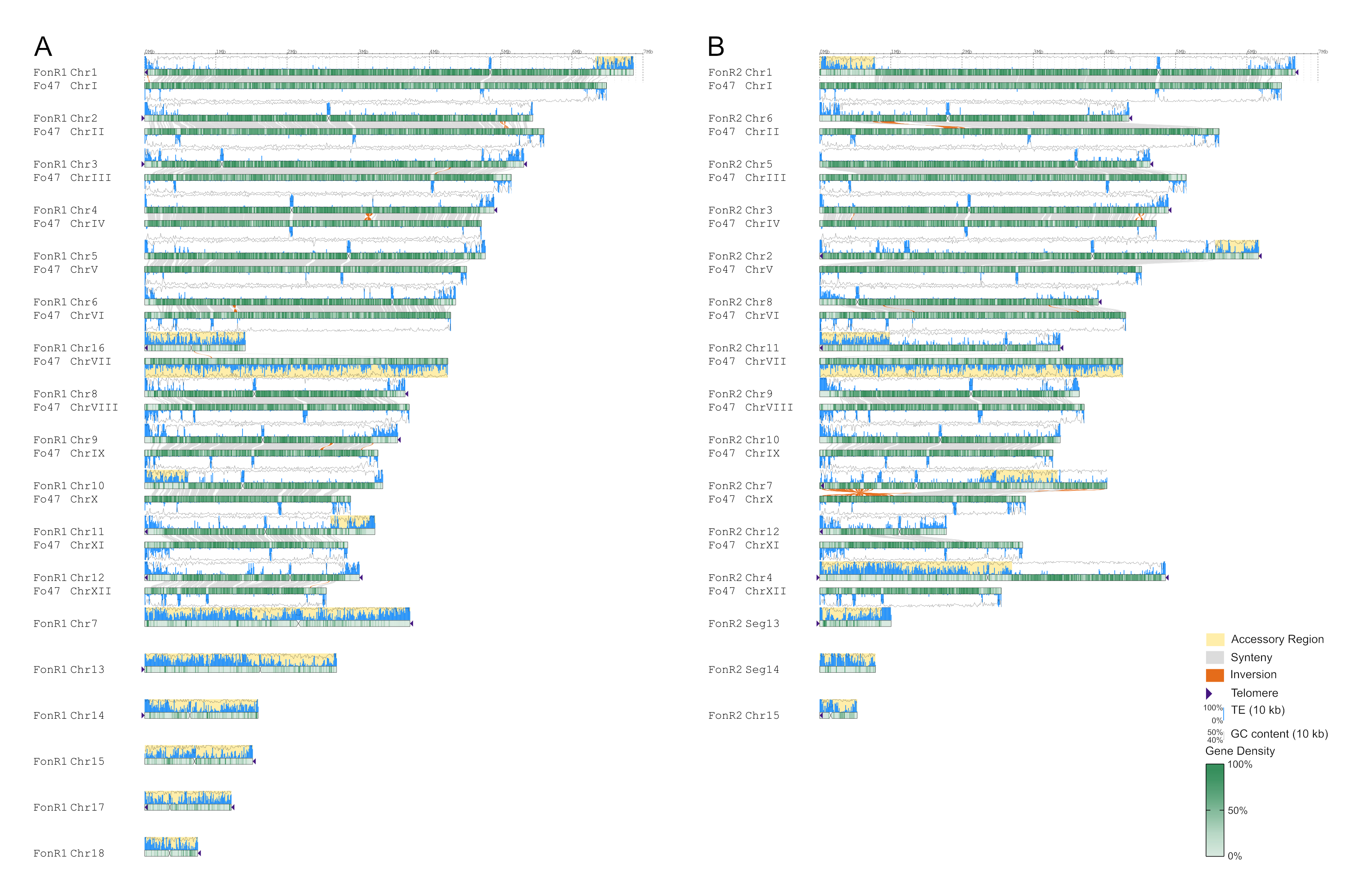

Supplement: S3 Fig — Ideograms of FonR1 (A) and FonR2 (B) were visualized using GenomeSyn [80]. Genomic features, including gene density, GC content, TE content, telomeres, centromeres, and genome synteny with Fo47 chromosomes are shown on the ideograms. The yellow background indicates the accessory regions. (TIFF) [file ppat.1013455.s003.tiff]

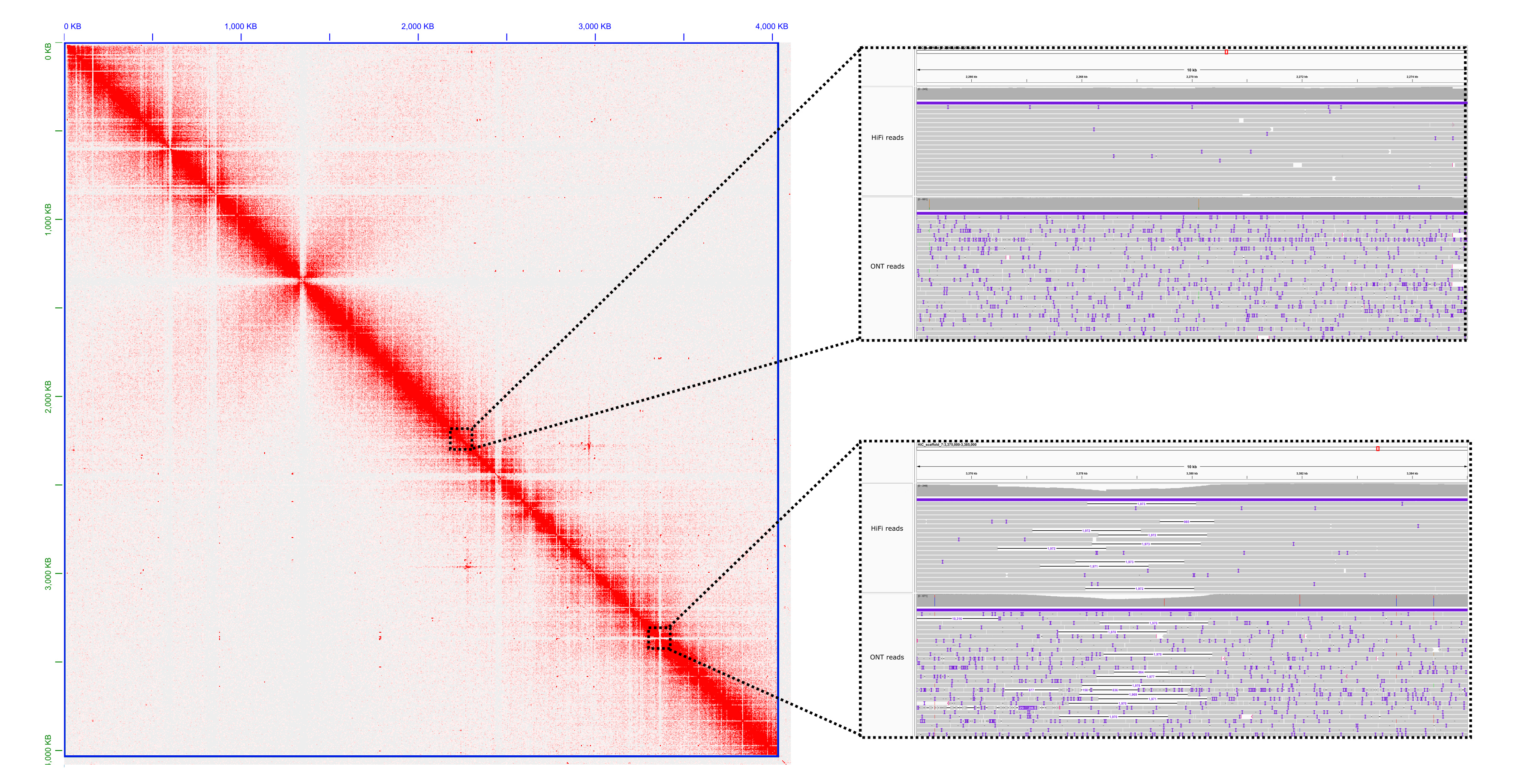

Supplement: S4 Fig — Hi-C contact map showing Chr07 of FonR2 on the left and IGV screenshots with HiFi and ONT reads mappings on the AR/CC borders. AR: accessory regions. CC: core chromosome. (TIFF) [file ppat.1013455.s004.tiff]

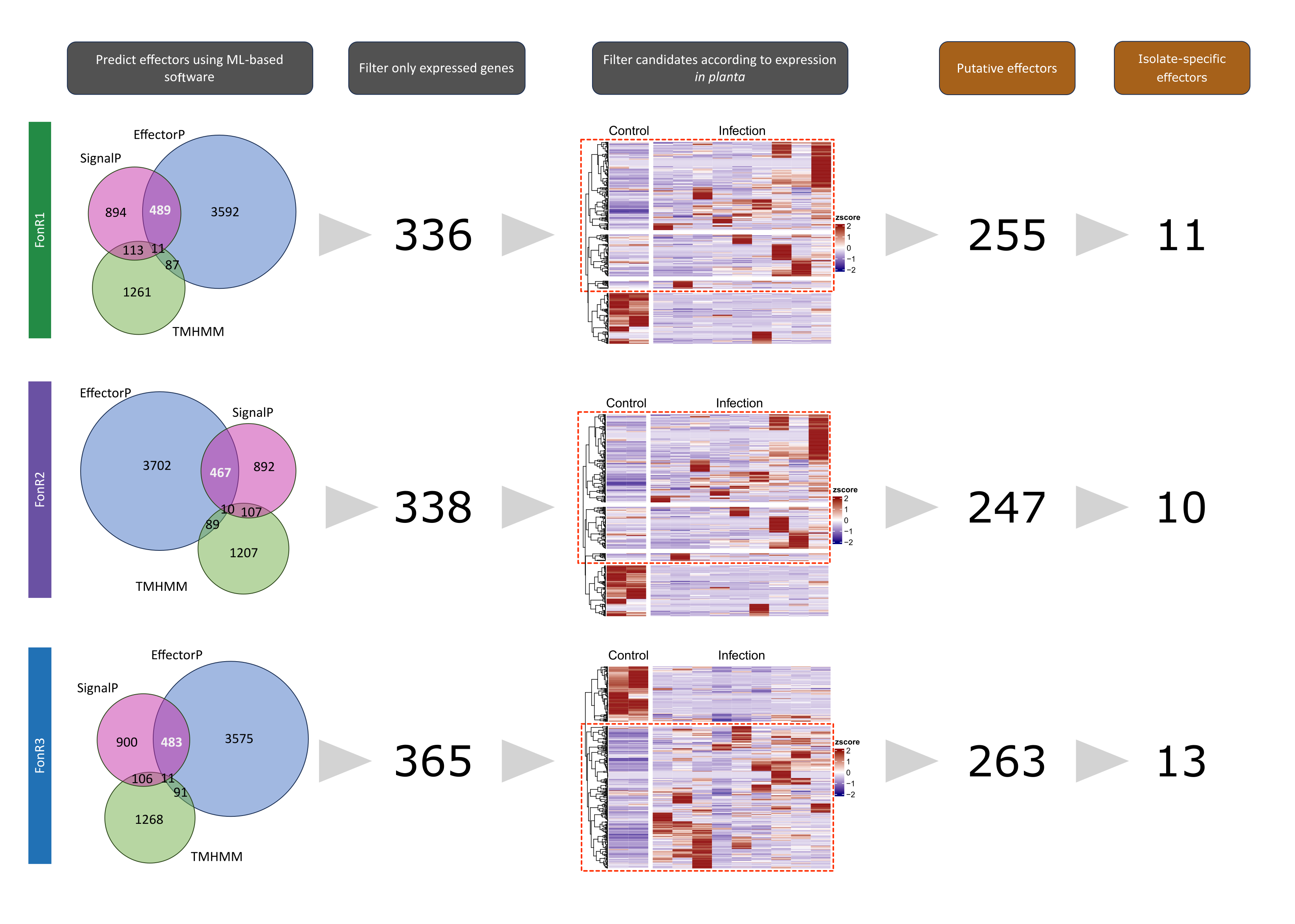

Supplement: S5 Fig — The effectors are first identified if they are predicted by SignalP [34] and EffectorP [33] but not including the transmembrane domain predicted by TMHMM [35]. After filtering, only the expressed genes in any of the selected conditions for transcriptome analysis, only genes that have increased expression in planta have been selected as putative effectors. Finally, isolate-specific effectors were identified with OrthoFinder [29]. (TIFF) [file ppat.1013455.s005.tiff]

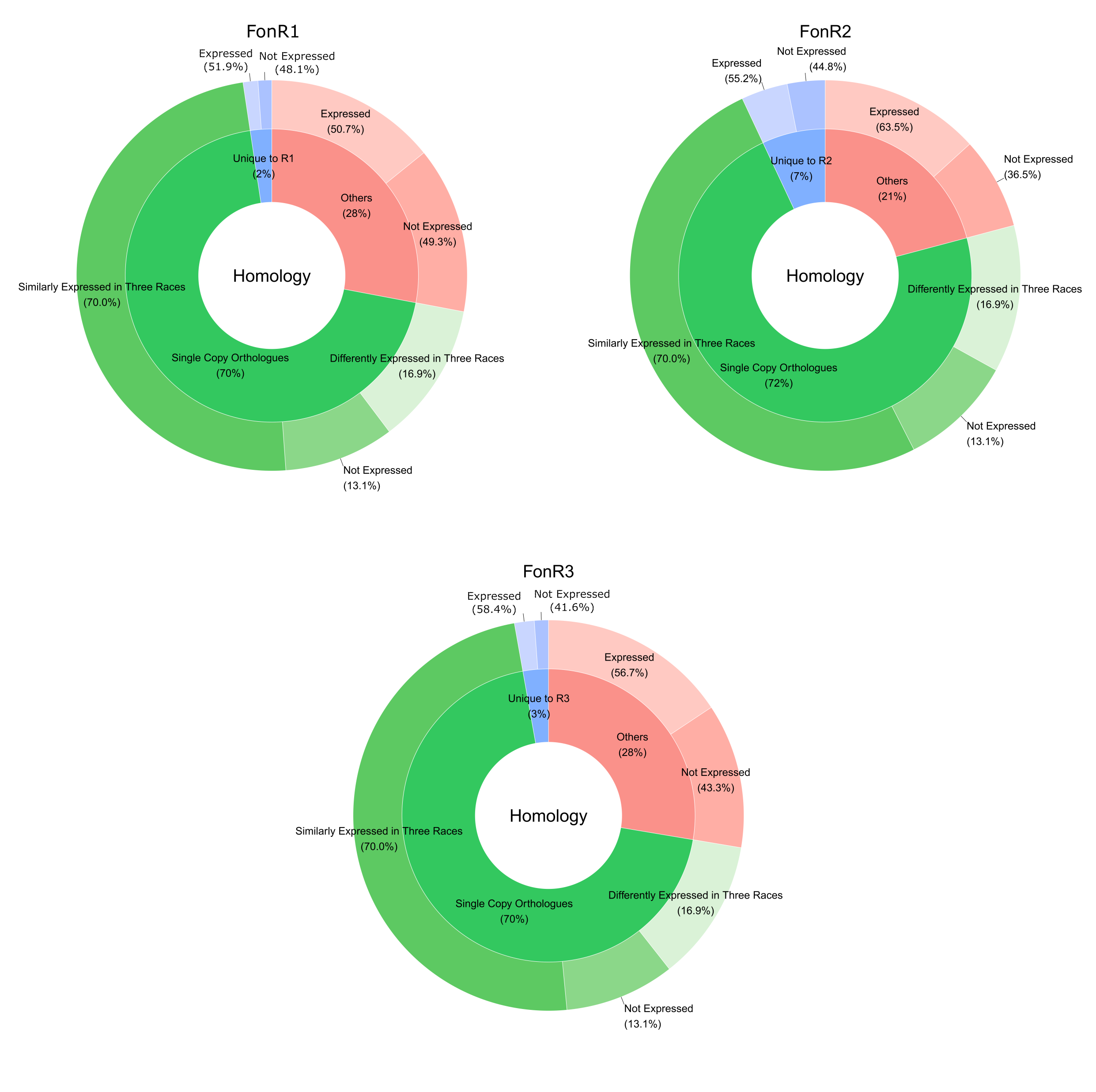

Supplement: S7 Fig — (TIFF) [file ppat.1013455.s007.tiff]

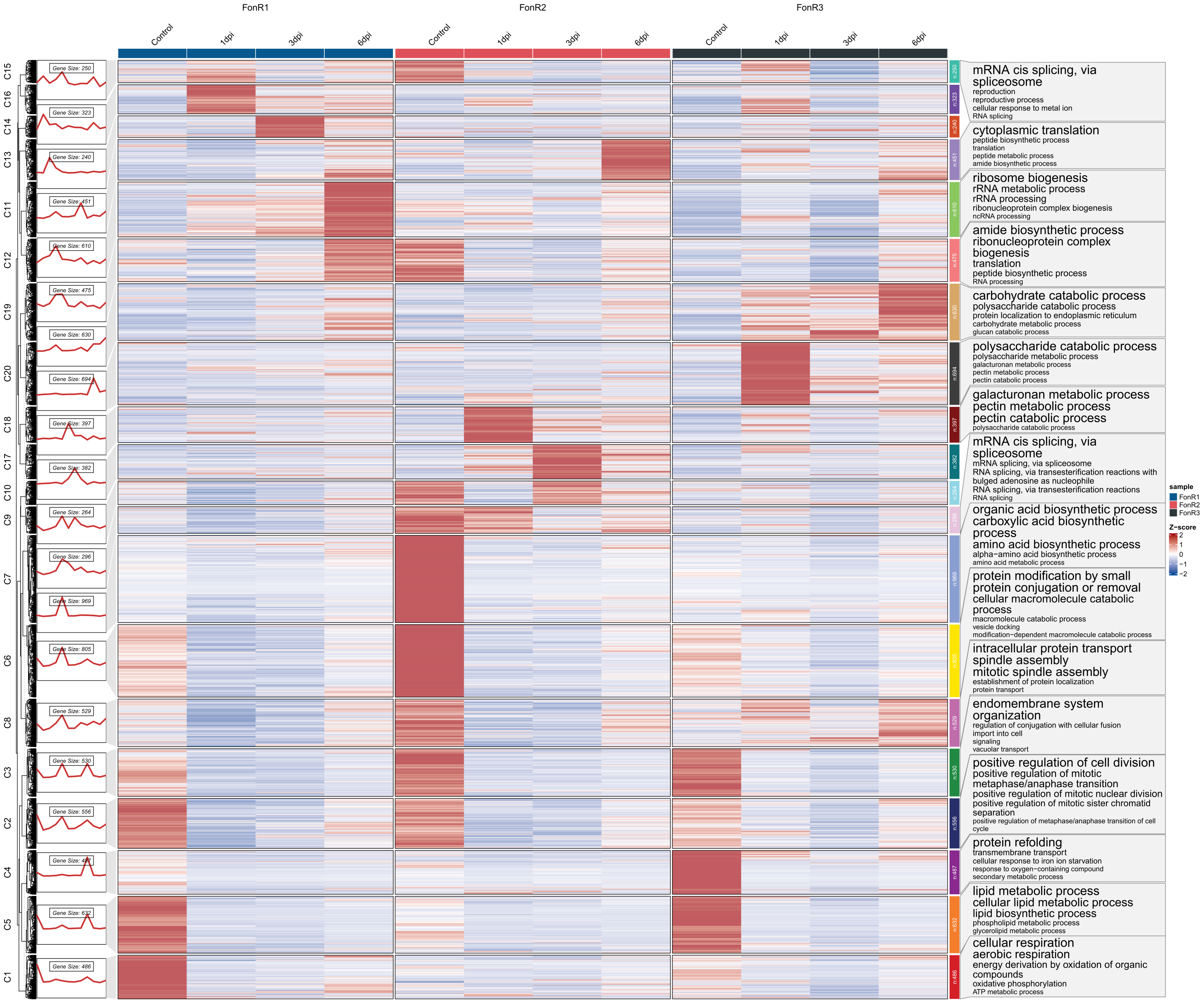

Supplement: S8 Fig — Significantly enriched gene ontology terms are listed on the right side. (TIFF) [file ppat.1013455.s008.tiff]

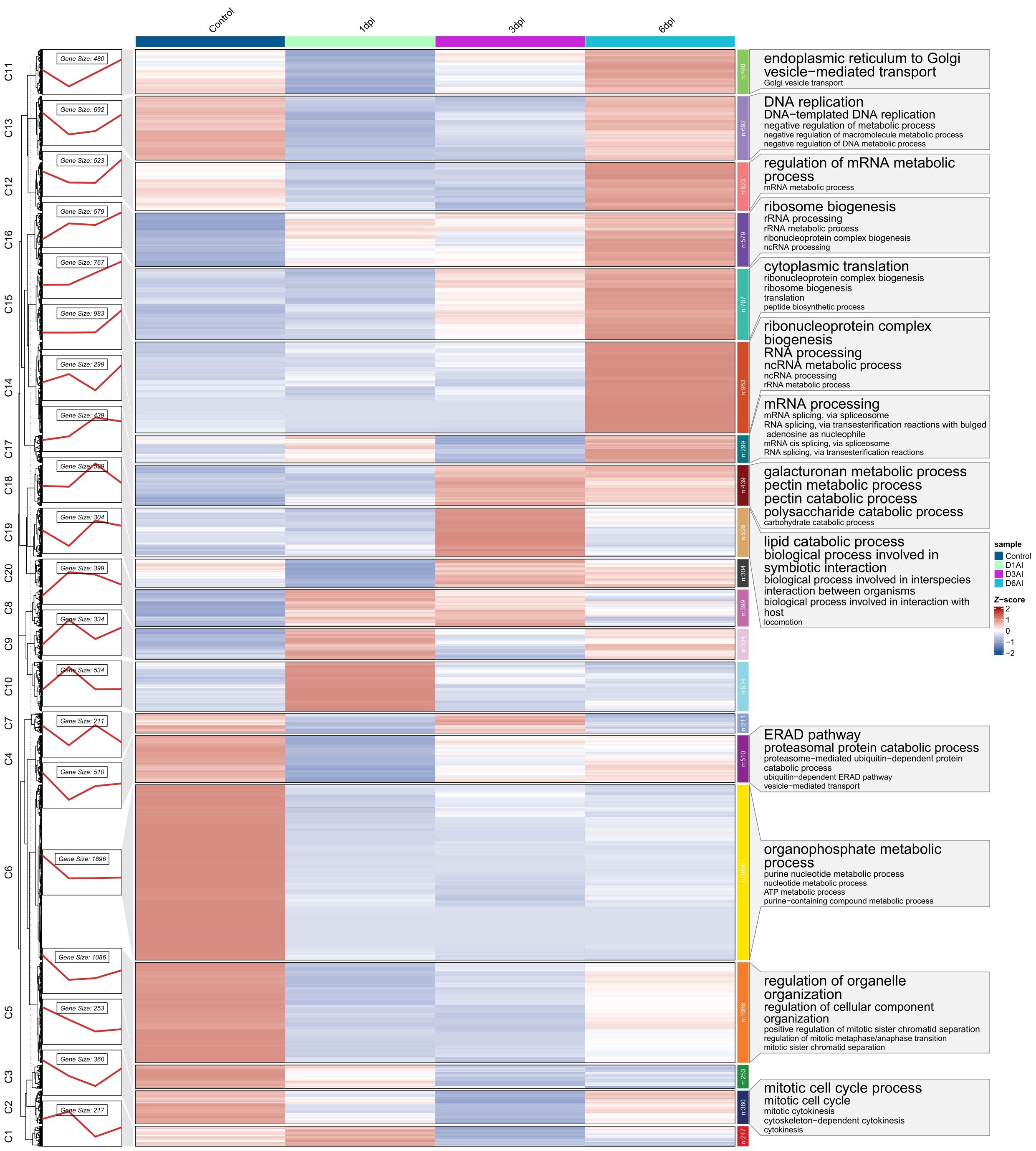

Supplement: S9 Fig — Significantly enriched gene ontology terms are listed on the right side. (TIFF) [file ppat.1013455.s009.tiff]

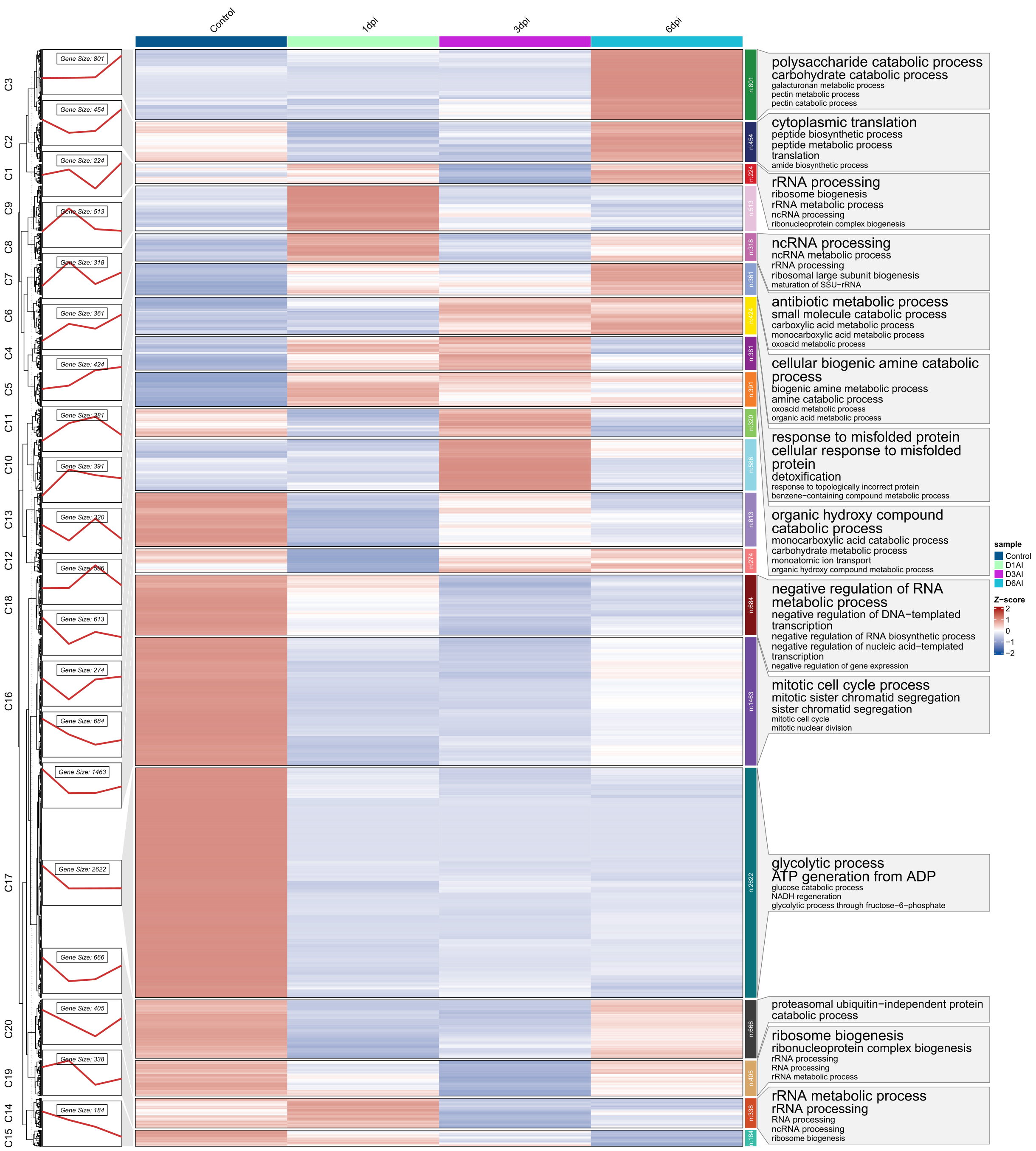

Supplement: S10 Fig — Significantly enriched gene ontology terms are listed on the right side. (TIFF) [file ppat.1013455.s010.tiff]

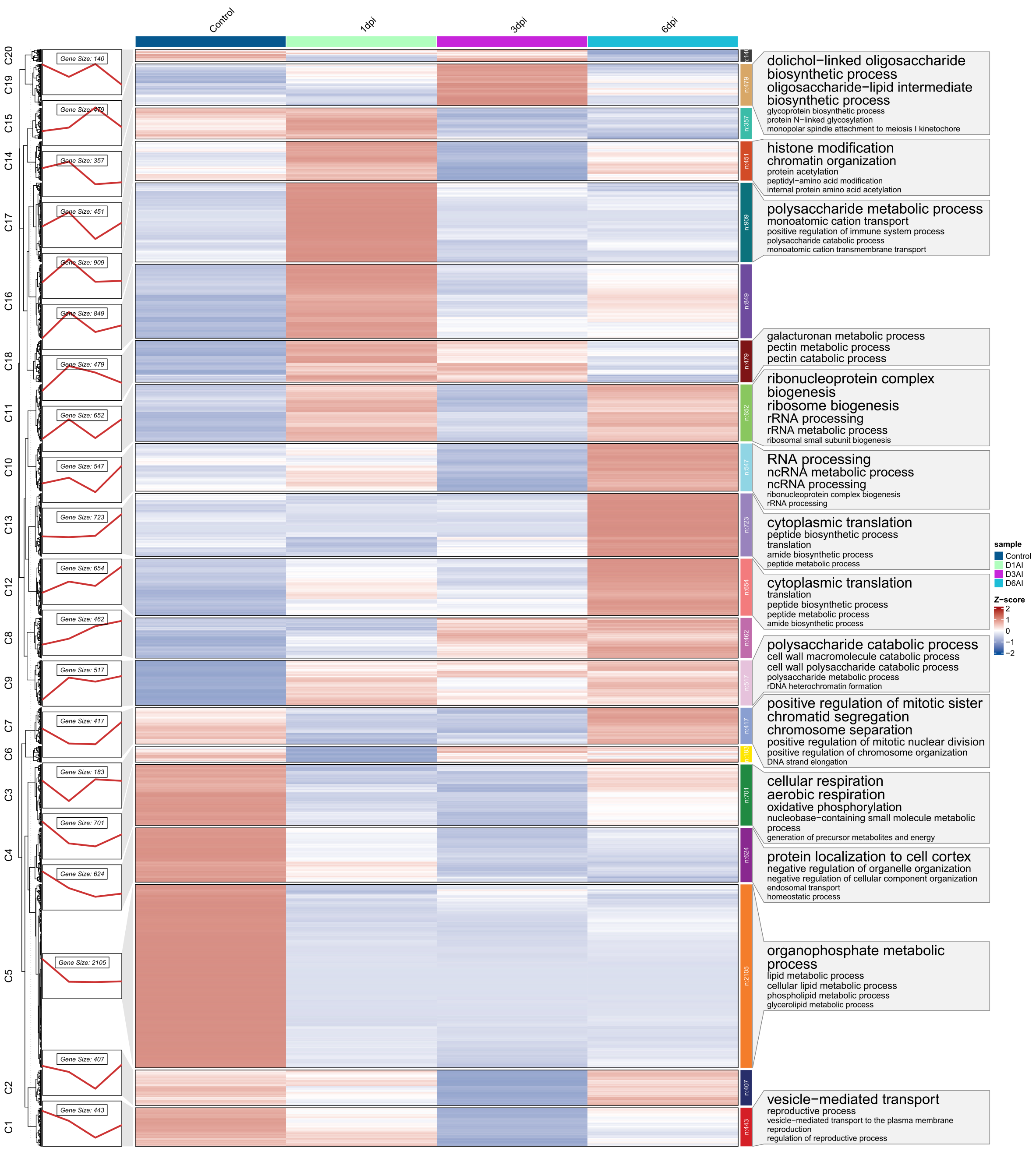

Supplement: S11 Fig — Significantly enriched gene ontology terms are listed on the right side. (TIFF) [file ppat.1013455.s011.tiff]

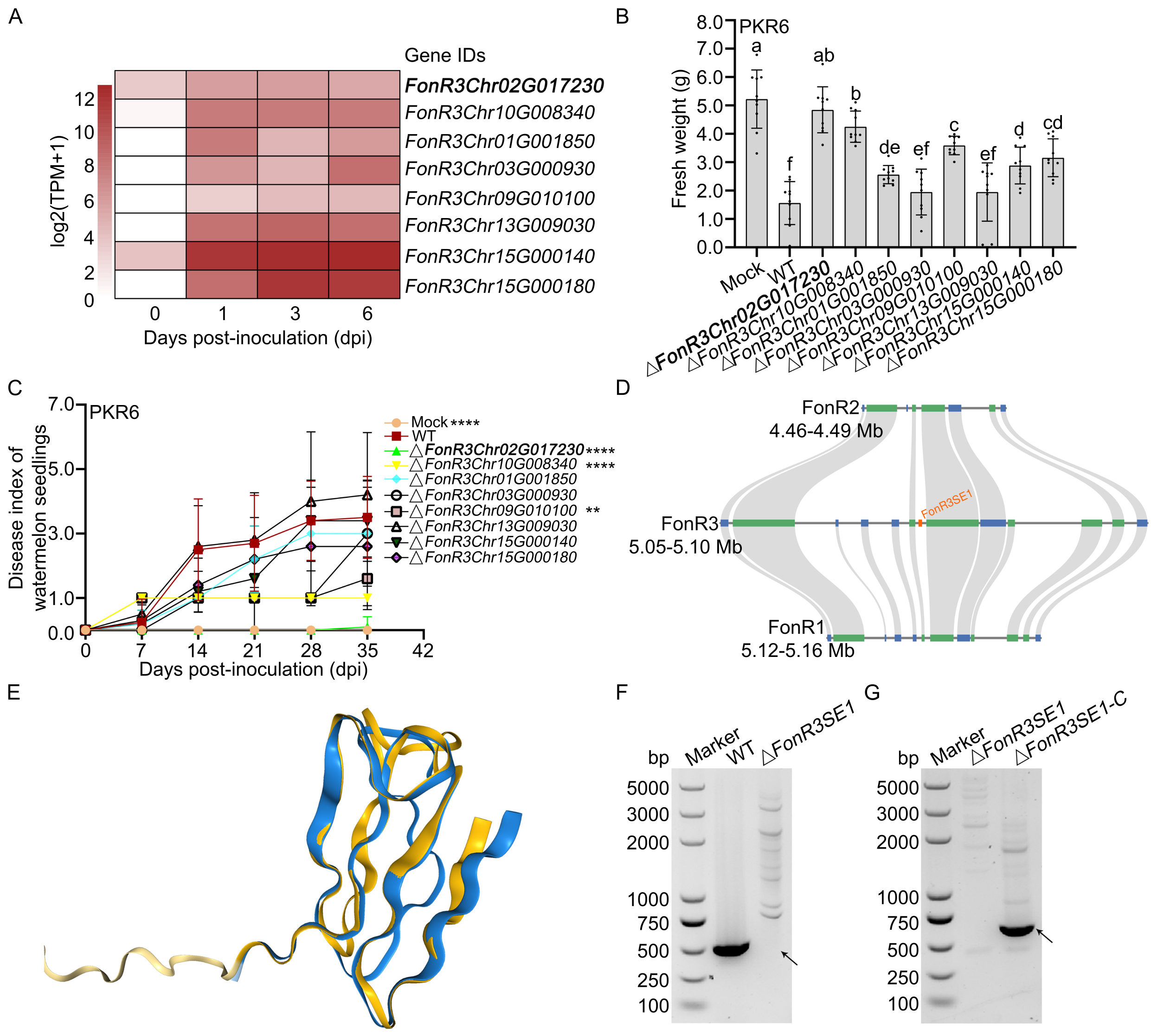

Supplement: S12 Fig — (A) The expression of eight putative FonR3-specific effectors in PDA medium (0 dpi) and during infection on cultivar G42 (1, 3, 6 dpi). (B, C) Fresh weights and disease indices of infected PKR6 seedlings by FonR3-specific effector mutants. For each treatment, 10 plants were used. Different letters indicate significant differences based on ANOVA analysis followed by Duncan’s multiple range test (p < 0.05). Disease index was evaluated based on a 7-scale rating: 0 = asymptomatic, 1 = slight stunted growth and yellowing, 3 = stunted growth and yellowing, 5 = wilting, 7 = dead. The Wilcoxon rank-sum test was applied to the disease indices between WT and mutants at 35 dpi. p-value < 0.01 **, p-value < 10-4 ****. Note: The FonR3Chr02G017230 gene was bold and given the name of FonR3SE1 in this study. (D) Microsynteny plot of the FonR3SE1 loci in FonR1, FonR2, and FonR3. Gray lines show syntenic blocks. Genes on the plus strand are shown in blue, while genes on the minus strand are shown in green. FonR3SE1 is shown in orange. (E) Superposition of FonR3SE1 predicted protein structure (blue) and Hirsutella minnesotensis 3608 Beta/gamma crystallin ‘Greek key’ domain containing protein (AF-A0A0F8A4Y3-F1-model_v4, yellow) generated by Foldseek [42]. (F-G) PCR detection of the FonR3SE1 gene in the WT, ΔFonR3SE1 (F), and ΔFonR3SE1-C (G). (TIFF) [file ppat.1013455.s012.tiff]

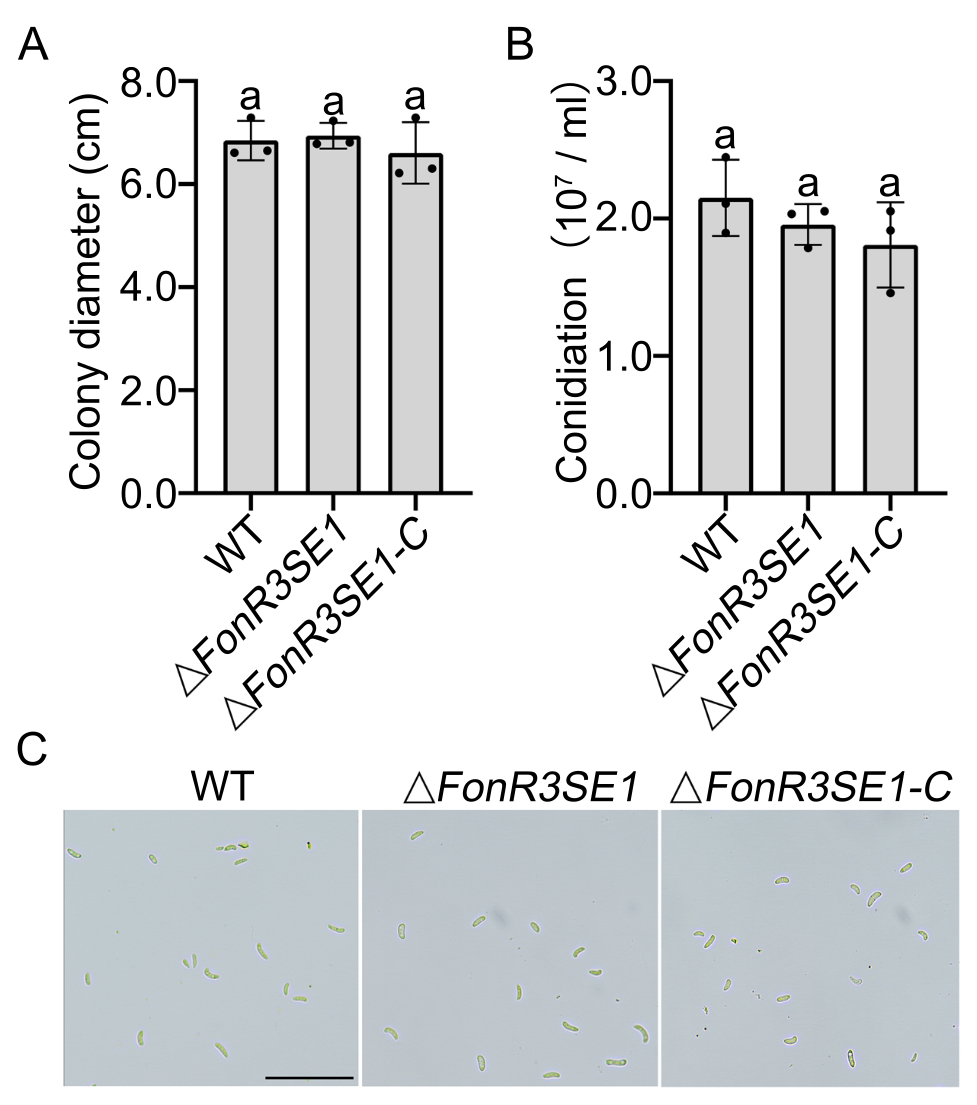

Supplement: S13 Fig — (A) Colony diameters of the WT FonR3, ΔFonR3SE1, and ΔFonR3SE1-C were measured on PDA plates at 25 °C at 5 dpi. (B) Conidial production was counted by using a hemocytometer at 3 dpi in PDB medium at 25°C in a 175-rpm shaker. (C) Conidial morphology at 3dpi. Scale bar = 50 μm. Mean and standard deviation (SD) of colony diameters and conidiation were calculated from three independent experiments. Different letters indicate significant differences based on one-way ANOVA analysis followed by Duncan’s multiple range test (p = 0.05). (TIFF) [file ppat.1013455.s013.tiff]

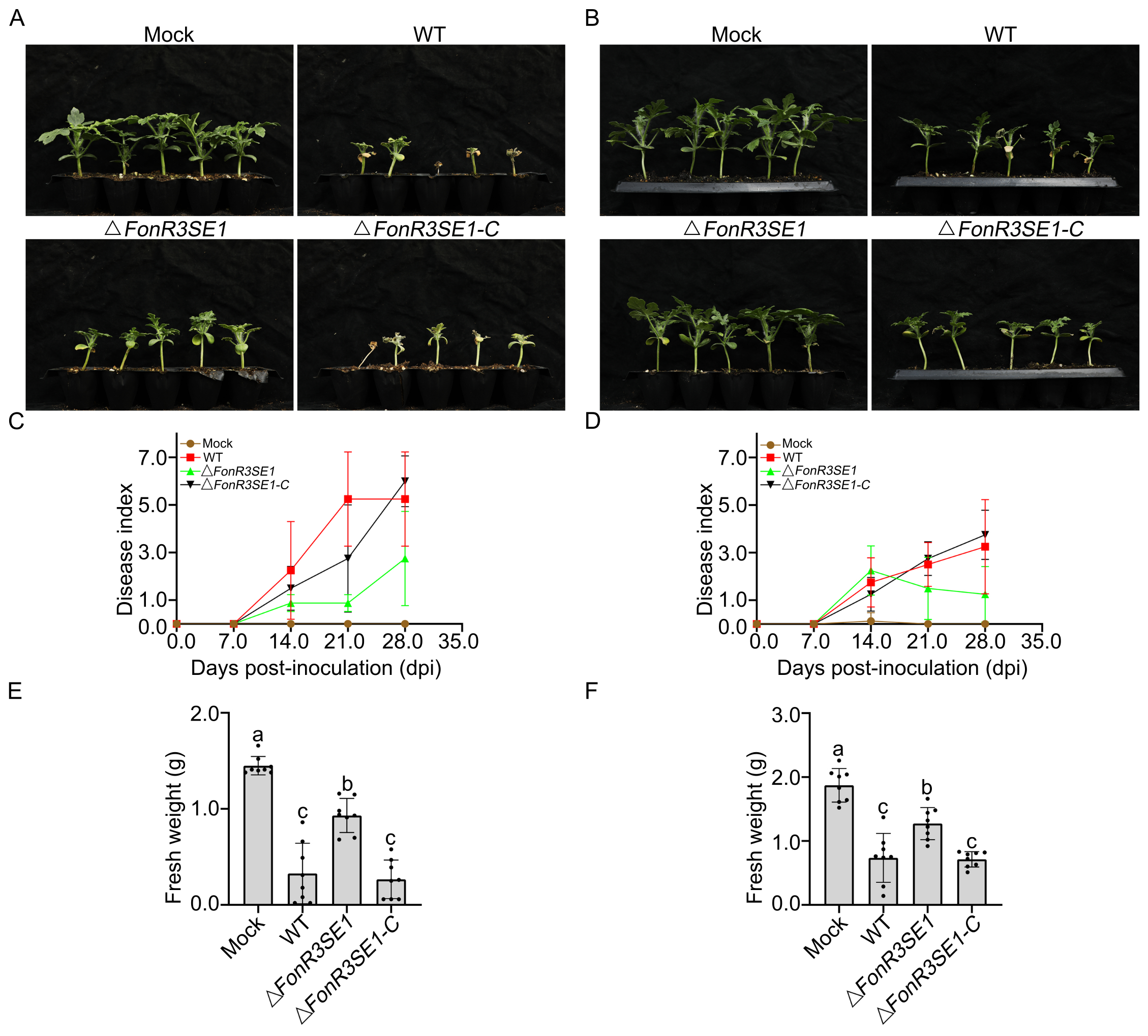

Supplement: S14 Fig — For each treatment, eight to ten plants were tested. (A-B) Infected PKR6 plants at 28 dpi using 11-day-old seedlings for inoculations. (C-D) The corresponding disease index of infected PKR6 seedlings at 28 dpi. (E-F) The corresponding fresh weights of the above-ground infected plants at 28 dpi. Different letters above the bars represent the significant differences between treatments using one-way ANOVA analysis followed by Duncan’s multiple range test (p = 0.05). (TIFF) [file ppat.1013455.s014.tiff]
